# Gems From the Caves: Genomic Insights Into the Biosynthetic Potential of Antimicrobial‐Producing Bacteria Associated With Tropical Cave‐Dwelling Sponges

**DOI:** 10.1111/1462-2920.70244

**Published:** 2026-01-21

**Authors:** Gabriel Rodrigues Dias, Bruno Francesco Rodrigues de Oliveira, Joana Sandes, Guilherme Muricy, Marinella Silva Laport

**Affiliations:** ^1^ Instituto de Microbiologia Paulo de Góes Universidade Federal do Rio de Janeiro Rio de Janeiro Brazil; ^2^ Departamento de Microbiologia e Parasitologia, Instituto Biomédico Universidade Federal Fluminense Niterói Brazil; ^3^ Departamento de Invertebrados, Museu Nacional Universidade Federal do Rio de Janeiro Rio de Janeiro Brazil

**Keywords:** antibacterial, biosynthetic gene clusters, Demospongiae, marine sponges, *Pseudomonadaceae*, submarine caves

## Abstract

Submarine caves are promising frontiers for novel biomolecules active against multidrug‐resistant bacteria. These habitats harbour rich communities of marine sponges, whose microbiomes produce diverse bioactive metabolites. The present study investigates the potential of bacteria isolated from cave‐dwelling marine sponges of the class Demospongiae of a tropical archipelago for the production of antimicrobial substances and reveals their biosynthetic diversity by genomic analyses. Ten out of the 89 antimicrobial‐producing strains showed inhibitory activity against Gram‐positive and Gram‐negative bacteria, encompassing strains with multidrug‐resistant phenotypes. Biosynthetic gene clusters (BGCs) encoding antimicrobial‐active metabolites were predicted in these sponge‐derived pseudomonads. Most BGCs exhibited low similarity (< 80%) with known clusters, indicating potential for novel metabolite discovery. Comparative genomics across Pseudomonadaceae genomes revealed both species‐specific and shared BGCs, including conserved clusters encoding for koreenceine and bokeelamides biosynthesis. Some cryptic BGCs encoded antimicrobial peptides (AMPs) together with proteins associated with maturation, regulation, immunity and export, suggesting roles in observed bioactivity. Altogether, this work expands the genomic and biosynthetic landscape of sponge‐associated Pseudomonadaceae and uncovers promising gene clusters for the biotechnological exploration of novel antimicrobials.

## Graphical abstract

Our study reveals that cave‐dwelling marine sponges host bacteria with antimicrobial activity against multidrug‐resistant strains. The bioactive Pseudomonadaceae strains exhibited biosynthetic clusters encoding both characterized and putative antimicrobial compounds and peptides. The findings highlight their potential for discovering novel biomolecules to combat multidrug‐resistant pathogens.

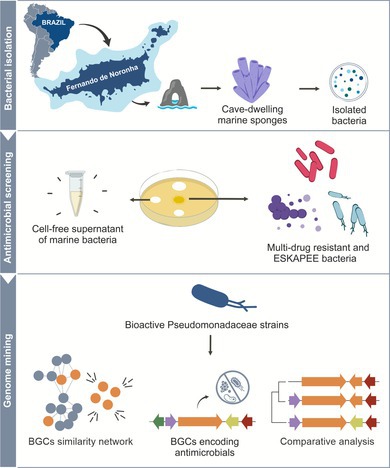

**Abbreviations**
AMR: antimicrobial resistance
ARTS: antibiotic resistant target seeker
BGCs: biosynthetic gene clusters
BHI: brain heart infusion
BHI 1: 10: BHI diluted ten times
BiG‐SCAPE: biosynthetic gene similarity clustering and prospecting engine
CFS: cell‐free supernatants
GBDP: genome BLAST distance phylogeny
GCFs: gene cluster families
GTDB: genome taxonomy database
KEGG: Kyoto encyclopedia of genes and genomes
KO: KEGG orthology
MALDI‐TOF: matrix‐assisted laser desorption/ionisation
Marine 1:10: marine ten‐fold diluted
MSM: minimal salt medium
NRPs: nonribosomal peptide synthetases
PKS: polyketide synthases
RiPPs: post‐translationally modified peptides
TYGS: type (strain) genome server
WHO: World Health Organization

## 1 Introduction

Antimicrobials are widely regarded as one of the most significant medical advancements in history (Hutchings et al. 2019). However, since their discovery, resistance has been observed for most antimicrobials, often emerging soon after or even before their introduction into clinical practice (Tang et al. 2023). An in‐depth analysis of global health impacts of antimicrobial resistance (AMR) over time revealed that over one million people died from AMR globally each year between 1990 and 2021. Future forecasts indicate AMR deaths will rise steadily in the coming decades, increasing by almost 70% by 2050 compared to 2022 (Naghavi et al. 2024). In response to this alarming trend, the World Health Organization (WHO) established in 2017 a priority list of antibiotic‐resistant pathogens to guide research and development efforts, which was recently updated. Among the critical priority pathogens are carbapenem‐resistant *Acinetobacter baumannii* and third‐generation cephalosporin‐resistant and carbapenem‐resistant Enterobacterales, which pose significant challenges in healthcare settings (Miller and Arias 2024; Tacconelli et al. 2018; WHO 2024). Still, each year, the total arsenal of effective antimicrobials is dwindling at an alarming rate, with few drugs under development and tested in the final stages of pre‐clinical and clinical trials (Butler et al. 2023; Gigante et al. 2024).

In light of the dearth of antimicrobial drugs in development, extreme environments emerge as a new frontier in the quest for novel biomolecules with wide‐ranging biotechnological and biomedical applications, aiming to reduce the threat of AMR (Wilson and Brimble 2009). One particularly intriguing of these extreme environments is submarine caves. The uniqueness of these habitats is largely due to limited light, oligotrophy and low water circulation (Gerovasileiou and Bianchi 2021). Few organisms are adapted to survive in such extreme conditions. Sponges have evolved different types of adaptations to thrive in the perpetual darkness and nutrient‐deficient conditions found in submarine caves, leading to a striking diversity of these animals in such ecosystems, which are still scarcely described to date (Muricy et al. 2024). These invertebrates are often found in strict association with microbial communities rich in specific metabolic routes and physiological capabilities, being able to produce a diverse array of bioactive metabolites (de Oliveira et al. 2023). In fact, submarine caves constitute ecological niches for well‐adapted microorganisms with a surprisingly high degree of biodiversity that often engage in diverse geological processes and ecological interactions and act as prolific sources of bioactive molecules (Kosznik‐Kwaśnicka et al. 2022). Combined, all these aspects lead to a higher probability of uncovering novel antimicrobial bioactive compounds from cave‐dwelling sponge‐associated bacteria (Lo Giudice and Rizzo 2022).

While in silico approaches are rapidly emerging as feasible ways to tackle marine diversity and overcome the problem of low culturability, the isolation of microorganisms still remains a fundamentally important step in addressing the bioprospecting of natural products (Rodrigues and de Carvalho 2022). Furthermore, delving into the genomic aspects of microorganisms enables the identification of putative biosynthetic gene clusters (BGCs) responsible for the production of bioactive molecules (Tran et al. 2019). Currently, associating cultivation‐dependent strategies and genome mining is at the cornerstone of the discovery of novel antimicrobial agents from microbial producers with potential applications in medicine and industrial settings (Sedeek et al. 2022).

Hence, the present study aims to explore the potential of bacteria isolated from marine sponges collected in submarine caves of a tropical archipelago in the Western Atlantic Ocean for the production of antimicrobial substances against antimicrobial resistant bacteria. We further investigate through bioinformatic analyses the genomic features of five bioactive Pseudomonadaceae strains, aiming to reveal their secondary metabolite biosynthetic potential and perform a comparative BGC similarity analysis, focused on unveiling the genomic signatures underlying the observed antimicrobial properties and broadening our understanding of the biosynthetic diversity and metabolic potential of sponge‐associated bacteria inhabiting marine cave environments.

## 2 Experimental Procedures

### 2.1 Sponge Samples and Collection Sites

Eighteen sponge samples belonging to eight species of class the Demospongiae (Table S1) were collected via SCUBA diving in two different caves in the Fernando de Noronha Archipelago (PE, NE Brazil): Sapata cave (3° 52ˊ S—32° 28ˊ W) at depths ranging 11.3–16.5 m and Ilha do Meio cave (3° 49′ 11.1ˊˊ S—32° 23′ 44.1ˊˊ W) at depths of 6.9–10.0 m. The water temperature at all sampling sites remained around 27°C–29°C. The identification of the sponges was performed through morphological and molecular analysis (28S rRNA and *cox*1 sequencing) as described (Muricy et al. 2024; Nascimento et al. 2025). The sponge samples (1.0–3.0 cm^2^) were kept in 10 mL sterile artificial seawater (ASW; % w/v: NaCl 2.34, MgSO_4_·7H_2_O 0.49, MgCl_2_·6H_2_O 0.4, CaCl_2_·H_2_O 0.15, KCl 0.075, NaHCO_3_ 0.017) supplemented with 1.0 μg/mL of amphotericin B (Sigma‐Aldrich, Missouri, USA) and transferred to the laboratory.

### 2.2 Bacterial Isolation

Each sponge sample was processed aseptically in 10 mL of ASW. The sample was initially macerated with a scalpel and then homogenised by vigorous agitation with glass beads until a near‐uniform suspension was obtained. For bacterial isolation, serial ten‐fold dilutions of these macerates were inoculated on the following isolation agar‐based media: Brain Heart Infusion (BHI) (Kasvi, Paraná, Brazil), BHI diluted ten times (BHI 1:10), Marine (Difco, Beirut, Lebanon), Marine ten‐fold diluted in deionised water (Marine 1:10), minimal salt medium (MSM; % w/v: NaCl 1.0, Na_2_HPO_4_ 0.5, KH_2_PO_4_ 0.2, MgSO_4_ 0.02, [NH_4_]_2_SO_4_ 0.2, and glucose 1.0) (Guimarães et al. 2021). All media were supplemented with amphotericin B (1.0 μg/mL) to prevent fungal growth. Plates were incubated at 28°C (±2°C) and monitored daily for bacterial growth for at least 7 days. Two to three colonies were selected by distinct morphology, purified from the primary culture, grown in their respective liquid culture media at 28°C (±2°C) under constant agitation (150 rpm), and stored at −80°C with 30% (v/v) glycerol (Isofar, Rio de Janeiro, Brazil) supplemented culture media.

### 2.3 Screening for Antibacterial Activity

To assess the inhibitory activity against bacterial indicator strains, cell‐free supernatants (CFS) of the sponge‐derived bacterial grown cultures (28°C/48 h) were obtained through centrifugation at 21,000 × *g* for 15 min at 4°C and tested in a spot‐over‐lawn assay (Canellas et al. 2024). Initially, approximately 1.5 × 10^8^ CFU/mL (0.5 McFarland scale) of *Staphylococcus aureus* ATCC 29213 dissolved in 0.85% saline (NaCl) solution were spread on BHI agar. Subsequently, 20 μL of each CFS were spotted onto BHI agar and the plates were incubated at 37°C for 24 h. Positive results for antimicrobial activity were considered based on visual observation of the inhibition zones around the spotted CFS. Sponge‐associated bacteria that inhibited *S. aureus* were further tested using the same method described above against 10 additional strains, including multidrug‐resistant (MDR) and pathogenic bacteria, some of which belong to the ESKAPEE group and are listed as priority pathogens by the WHO for AMR (Miller and Arias 2024; WHO 2024) (Table S2). All tests were performed in triplicate and sterile culture medium was used as a negative control.

### 2.4 Identification of Bioactive Strains and Assessment of Clonal Relationships

Each marine bacterial strain presenting antimicrobial activity was submitted for identification by Matrix‐Assisted Laser Desorption/Ionisation (MALDI‐TOF) mass spectrometry (MS) on the Microflex LT MS platform (Bruker Daltonics, Massachusetts, EUA) (Rodrigues et al. 2017). The mass spectra obtained were compared with references in the database using MALDI Biotyper 4.1.100 (Bruker). The scoring values adopted for identification were those recommended by the manufacturer: 2.00–3.00 indicates great reliability (confidence for the species); 1.700–1.999 indicates low reliability identification (confidence for genus only); and < 1.6900 considered unreliable for identification.

BOX‐PCR was eventually used to estimate the relative degrees of similarity among selected marine strains with antimicrobial activity from the same species and to help determine whether strains are clonally related according to Versalovic (1994).

### 2.5 Whole Genome Sequencing and Assembly

Genomic DNA was extracted from each sponge‐associated Pseudomonadaceae strain presenting antimicrobial activity grown for 24 h on BHI‐agar using the Wizard Genomic DNA Purification Kit (Promega, Wisconsin, USA). DNA was quantified on the NanoDrop Lite (Thermo Scientific, Massachusetts, EUA) spectrophotometer. Libraries were prepared with a Nextera XT Kit (Illumina, California, EUA), purified with Agencourt AMPure XP beads (Beckman Coulter, California, USA), and quantified by qPCR (KAPA Fast Universal kit—Illumina/Universal). Whole‐genome sequencing was then performed on the Illumina NextSeq 500 System, with the NextSeq 500 MID Output kit (300 cycles). Sequencing quality was assessed with FastQC v0.11.9 (Andrews, 2010). Raw reads were processed with Trimmomatic v0.39 and assembled using SPAdes v3.13.1 (Bankevich et al. 2012). Genome quality was evaluated with assembly‐stats v1.0.1 (https://github.com/sanger‐pathogens/assembly‐stats), seqkit v2.1.0 (Shen et al. 2024), and CheckM2 v1.0.2 (Chklovski et al. 2023). Annotation was performed with Prokka v1.14.6 (Seemann 2014), and functional characterisation with EggNOGG‐mapper v2.1.12 using the EggNOGG v5.0 database with SMART protein domain annotation (Cantalapiedra et al. 2021; Huerta‐Cepas et al. 2019; Letunic et al. 2021). Kyoto Encyclopedia of Genes and Genomes (KEGG) Orthology (KO) assignment and KEGG mapping were performed with BLASTKOALA (Kanehisa et al. 2016) and KEGG mapper tool (Kanehisa and Sato 2020; Kanehisa et al. 2022), respectively. Genomes were deposited at NCBI under the Bioproject PRJNA1207527 (Table S3).

### 2.6 Genome‐Based Taxonomy and Phylogenomics Analysis

Whole genome‐based taxonomic analysis was performed with the Genome Taxonomy Database (GTDB) toolkit (GTDB‐Tk v2.4.0) using the GTDB database release R220 (Chaumeil et al. 2022; Parks et al. 2022). Genome sequence data were uploaded to the Type (Strain) Genome Server (TYGS) for phylogenomic inferences (Meier‐Kolthoff and Göker 2019). Pairwise comparisons were conducted using genome BLAST distance phylogeny (GBDP) and accurate intergenomic distances inferred under the algorithm ‘trimming’ and distance formula d5 (Meier‐Kolthoff et al. 2013), with 100 distance replicates calculated for each analysis. Digital DDH values and confidence intervals were calculated using the recommended settings of the GGDC 4.0 (Meier‐Kolthoff et al. 2022). The resulting intergenomic distances were used to infer a balanced minimum evolution tree with branch support that was inferred from 100 pseudo‐bootstrap replicates each via FASTME 2.1.6.1 including SPR postprocessing (Farris 1972; Lefort et al. 2015). The trees were rooted at the midpoint and visualised with PhyD 3 (Kreft et al. 2017). The average nucleotide identity (ANI) of the genomes was determined using OrthoANI software (Lee et al. 2016).

### 2.7 Biosynthetic Gene Cluster Annotation and Analysis

BGCs were identified using DeepBGC (v0.1.31) (Hannigan et al. 2019) with score threshold set to 0.7 and minimum number of known biosynthetic protein domains in a BGC set to 1. The resulting JSON files and genomic sequences were further uploaded and analysed with antiSMASH 8 (v8.0.4) (Blin et al. 2023) with default parameters and all extra features set to on. DeepBGC candidates were further filtered for (i) being categorised with a known product class, (ii) having more than one identified gene, (iii) presenting length > 2 kbp and (iv) containing at least one known biosynthetic pfam or TIGRFAM protein domain, according to Gounot et al. (2022). BGCs further classified as ‘unknown’ were required to have < 80% similarity to any existing sequence in the antiSMASH 8.0 and MIBiG v3.1 databases using the KnownClusterBlast and ClusterBlast results from antiSMASH 8.0. Additionally, antiSMASH 8.0 output.gbk files were submitted to Antibiotic Resistant Target Seeker v2.0 (ARTS; https://arts.ziemertlab.com) (Mungan et al. 2020) in order to provide genomic context to the clusters, such as proximity to duplicated core genes and known antibiotic‐resistance genes. LoVis4u v0.1.5 was employed to analyse synteny between different BGC regions (Egorov and Atkinson 2025). Pairwise sequence alignment of protein sequences was done with EMBOSS Needle within EMBL‐EBI Job Dispatcher sequence analysis tools framework (Madeira et al. 2024). To uncover putative antimicrobial peptides (AMPs), AMPGram (Burdukiewicz et al. 2020) and AMPv2Analyzer (Veltri et al. 2018) were employed in conjunction and only those CDS with scores > 0.5 in both tools were considered positive.

### 2.8 Biosynthetic Gene Cluster Networks

To further explore BGC diversity, genomes of each species (*P. juntendi*, *P. asiatica* , and *E. khazarica*) available in the National Center for Biotechnology Information (NCBI) database (accessed on August 24, 2025) were retrieved and analysed using the same workflow described above. Only high‐quality genomes (> 99% completeness and < 1% contamination) were included. For *P. juntendi* and *P. asiatica* , additional genomes were selected to total ten per species, while only six high‐quality *E. khazarica* genomes from different studies were available, totaling eight analysed genomes (Table S4). The BGCs were grouped into gene cluster families (GCFs) by sequence similarity using the Biosynthetic Gene Similarity Clustering and Prospecting Engine (BiG‐SCAPE) v1.1.5 with clusters from MIBiG database v3.0 (Navarro‐Muñoz et al. 2020). Gene cluster diagrams of BGCs were visualised using Cytoscape v3.10.3 (Shannon et al. 2003).

## 3 Results

### 3.1 Bacterial Symbiont Isolation and Screening for Antibacterial Activity

**FIGURE 1:**
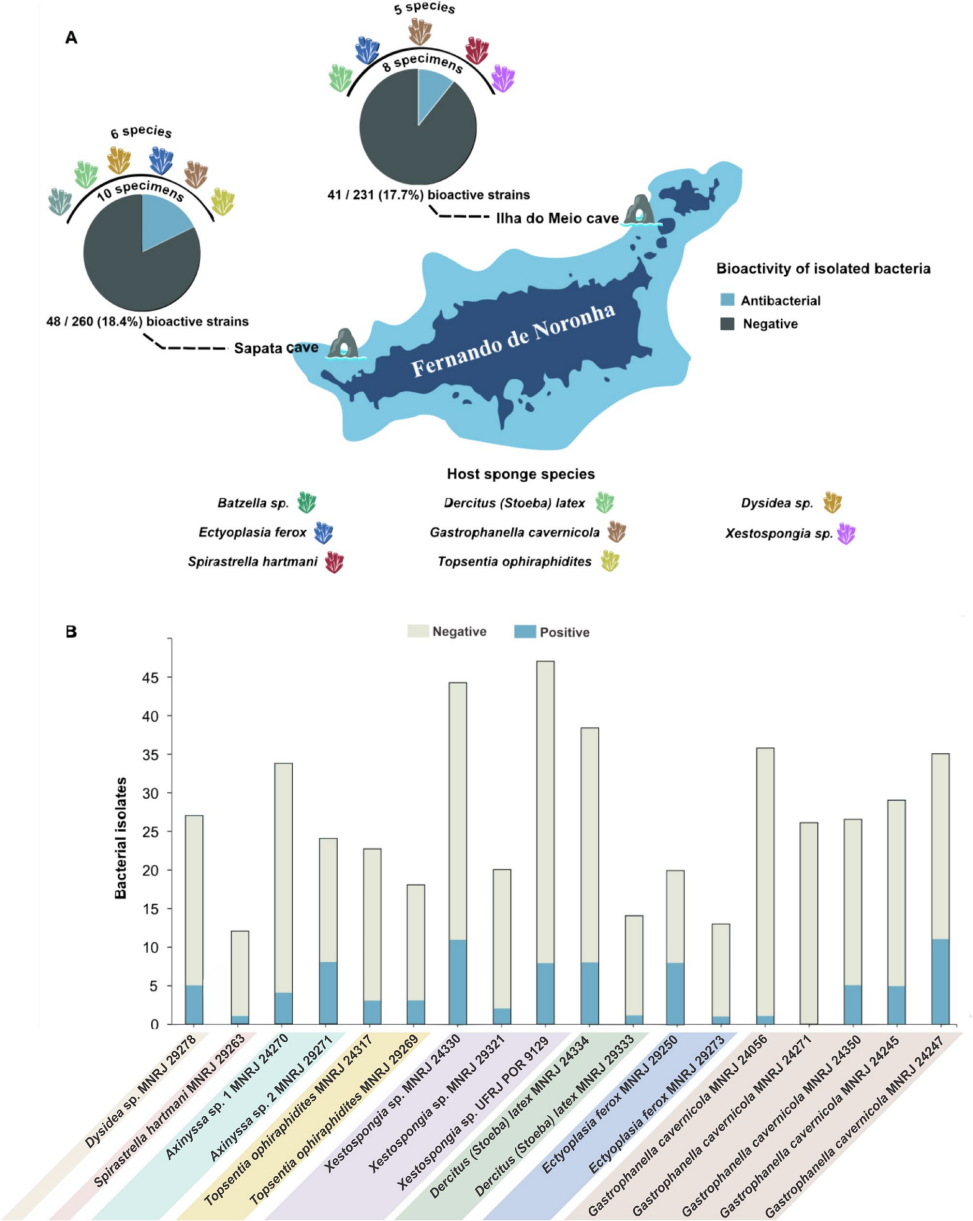
Antimicrobial activity screening of the cell‐free supernatant (CFS) of the cave‐dwelling sponge‐associated bacteria against *Staphylococcus aureus* ATCC 29213. (A) Bacterial isolation and profile of antimicrobial activity in strains isolated in relation to collection sites in the Fernando de Noronha Archipelago (PE, NE Brazil). The taxonomic affiliation, at the genus and species level, of collected and host sponges for bacterial isolation is represented as icons of different colours. The total number of specimens of collected sponges is shown just above the pie charts, while below is the relationship between the total number of bioactive strains isolated from the respective specimens. The legend on the left defines colour codes for tracking the antibacterial activity in blue and grey for none. (B) Proportion of bioactive bacterial strains from each sponge sample (in blue). Sponges are grouped according to their species or genus in different colours.

A total of 491 marine bacteria were isolated from the 18 Demospongiae samples. In the initial screening, 89 (18.1%) out of 491 isolates inhibited the growth of *S. aureus* ATCC 29213. Of these, 260 bacterial strains were isolated from 10 sponge specimens collected in Sapata cave and 231 strains were isolated from eight specimens sampled in Ilha do Meio cave (Figure 1A). Notably, the proportion of antibacterial‐bioactive isolates was comparable between both sites. Except for *G. cavernicola* MNRJ 24271, all sponge specimens yielded at least one bioactive strain (Figure 1B). Notably, the sponges *Xestospongia* sp. MNRJ 24330 (Ilha do Meio cave) and *G. cavernicola* MNRJ 29247 (Sapata cave) stood out, providing the highest number of bioactive strains (*n* = 10 and *n* = 11, respectively), which accounted for 22.2% and 30.5% of the bacteria isolated from these particular sponge samples.

Nine genera and 12 species belonging to the phyla Pseudomonadota and Bacillota were detected in the bioactive strains through MALDI‐TOF MS, of which *Vibrio* (48.3%) and *Pseudomonas* (12.3%) were the most prominent genera representatives. In particular, *Vibrio alginolyticus* (40.4%) and *Vibrio harveyi* (10.1%) were the top representatives at the species level (Figure S1).

### 3.2 Antibacterial Activity Against Multidrug‐Resistant Bacteria

**FIGURE 2:**
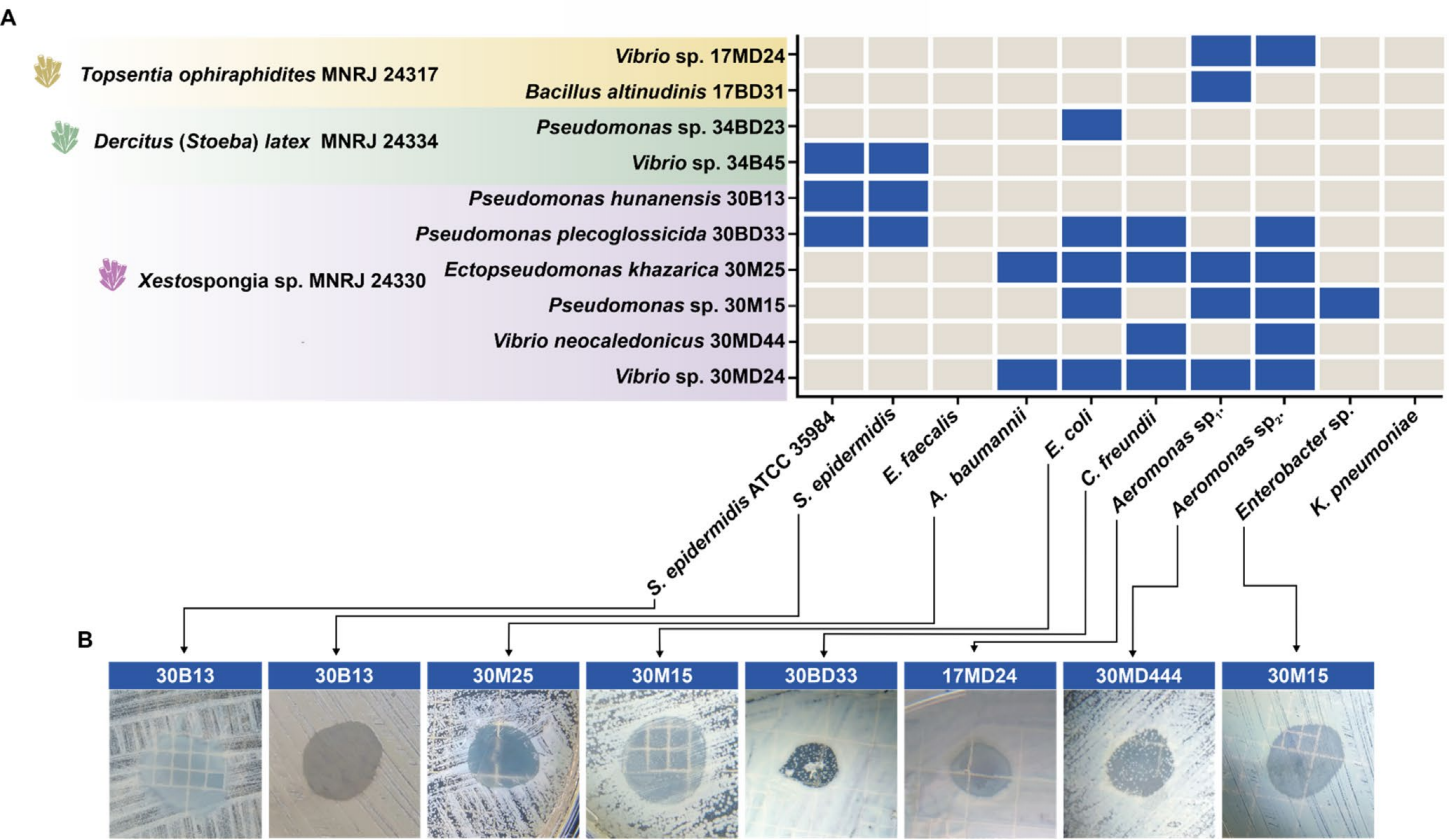
Antibacterial activity of the most promising sponge‐associated bacterial strains against other antimicrobial‐resistant strains of clinical and environmental origin. Strains are grouped according to the sponge host from which they were isolated. Main inhibition halos against each indicator strain are also represented. *S. epidermidis* ATCC 35984 producing biofilm; *S. epidermidis* resistant to beta‐lactams, quinolones and tetracyclines (MDR); *E. faecalis* resistant to streptomycin; *A. baumannii* resistant to oxacillin; *C. freundii* resistant to aminoglycosides, beta‐lactams, macrolides and quinolones (MDR); *Aeromonas* sp1. resistant to colistin and carbapenem; *E. coli* resistant to quinolones; *Aeromonas* sp2. resistant to colistin, beta‐lactams and mercury; *Enterobacter* sp. resistant to quinolones; *K. pneumoniae* resistant to beta‐lactams, fluoroquinolones and aminoglycosides (MDR).

Among the 89 positive strains in the antibacterial screening, ten also inhibited the growth of other Gram‐positive and Gram‐negative indicator strains of clinical and environmental origin (Figure 2). Interestingly, most of the strains (6 out of 10) were isolated from one single sponge, *Xestospongia* sp. MNRJ 24330, while two were obtained from *Topsentia ophiraphidites* MNRJ 24317 and two from *Dercitus (Stoeba) latex* MNRJ 24334. Noteworthy, *Vibrio* sp. 34B45 and *Pseudomonas hunanensis* 30B13 exhibited antimicrobial activity only against the Gram‐positive indicator strains of *S. epidermidis* . On the other hand, *Pseudomonas plecoglossicida* 30BD33, *E. khazarica* 30M25 and *Vibrio* sp. 30MD24 presented the widest activity spectrum, inhibiting the growth of five indicator strains tested, including MDR *S. epidermidis* , *Acinetobacter baumannii* resistant to oxacillin, *Escherichia coli* resistant to quinolones, MDR *Citrobacter freundii* and *Aeromonas* sp. resistant to colistin and beta‐lactams. It is also worth highlighting the strain *Pseudomonas* sp. 30M15, which was the only one capable of inhibiting *Enterobacter* sp. with quinolone resistance.

### 3.3 Genome Sequencing and Phylogenetic Analysis

Given the prominence of Pseudomonadaceae strains with antimicrobial activity and the confirmation of its non‐clonal relationship by BOX‐PCR analysis (Figure S2), their genome sequencing was performed to further investigate phylogenetic diversity and biosynthetic potential. Overall, the genomes varied from 5.29 to 5.92 Mbp in size with GC contents of 62.2% to 64.7%. An average of 3705 CDS, four rRNA, and 40 tRNA‐coding genes were assigned, with an estimated coding ratio of 88.7% (Table S5). All genomes contained at least one CRISPR system and one Cas protein.

**FIGURE 3:**
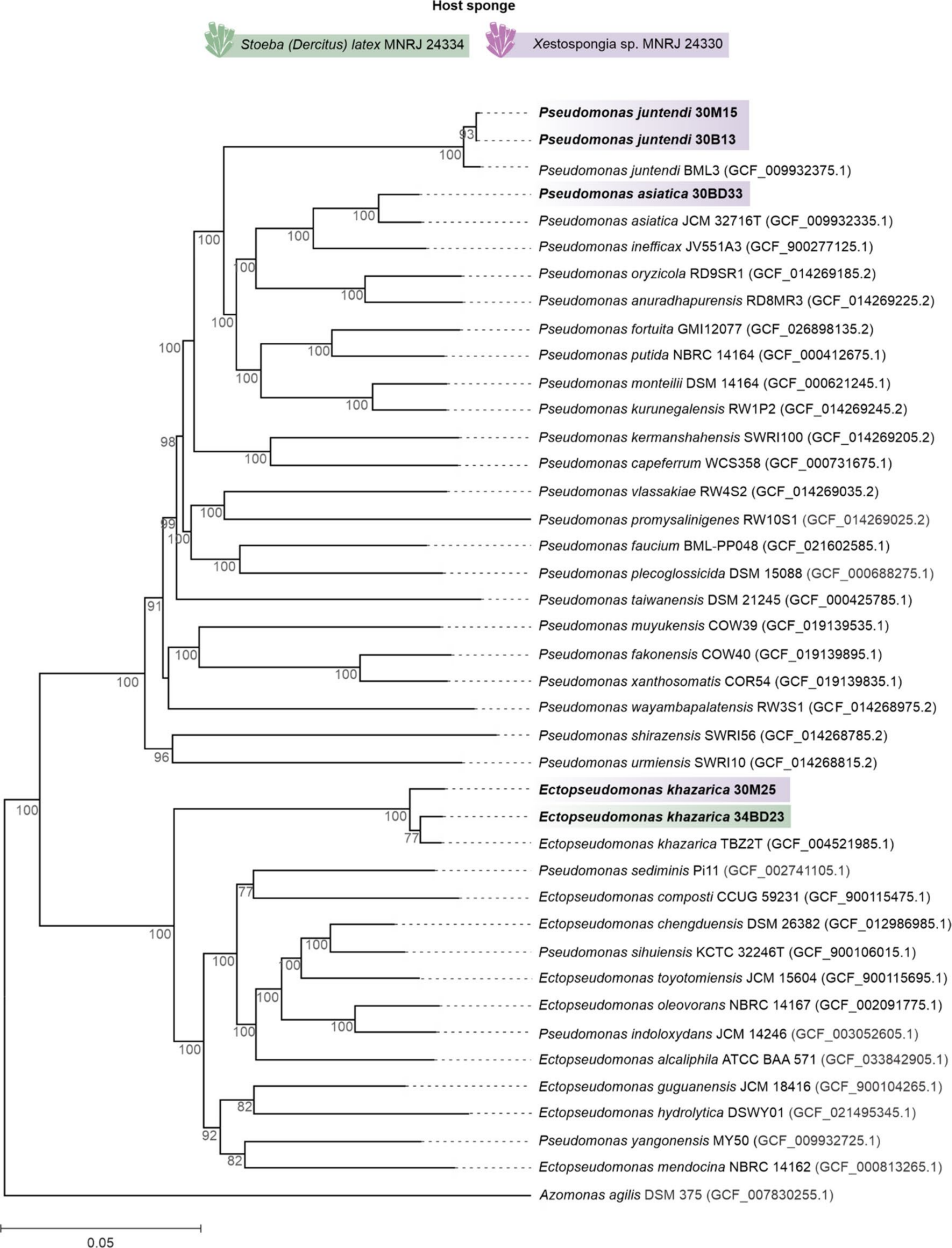
Phylogenetic tree inferred with FastME 2.1.6.1 from GBDP distances calculated from genome sequences. Tested strains are shown in bold. The numbers below the branches are GBDP pseudo‐bootstrap support values from 100 replications, with an average branch support of 72.5%. The tree was rooted at the midpoint. *Azomonas agilis* DSM 375 (GCF_007830255.1) was included as an external group. Strains of this study are highlighted in bold.

The strains exhibited an ≥ 97.64% ANI and therefore were identified at the species level (Table S3). Based on whole genome phylogenetic analyses, the five strains clustered into three clades within the Pseudomonadaceae family and within the genera *Pseudomonas* and *Ectopseudomonas* close to reference genomes of their species as presented in Figure 3. Only *E. khazarica* 30M25 maintained the preliminary identification by MALDI‐TOF MS.

### 3.4 Biosynthetic Gene Cluster (BGC) Mining

**FIGURE 4:**
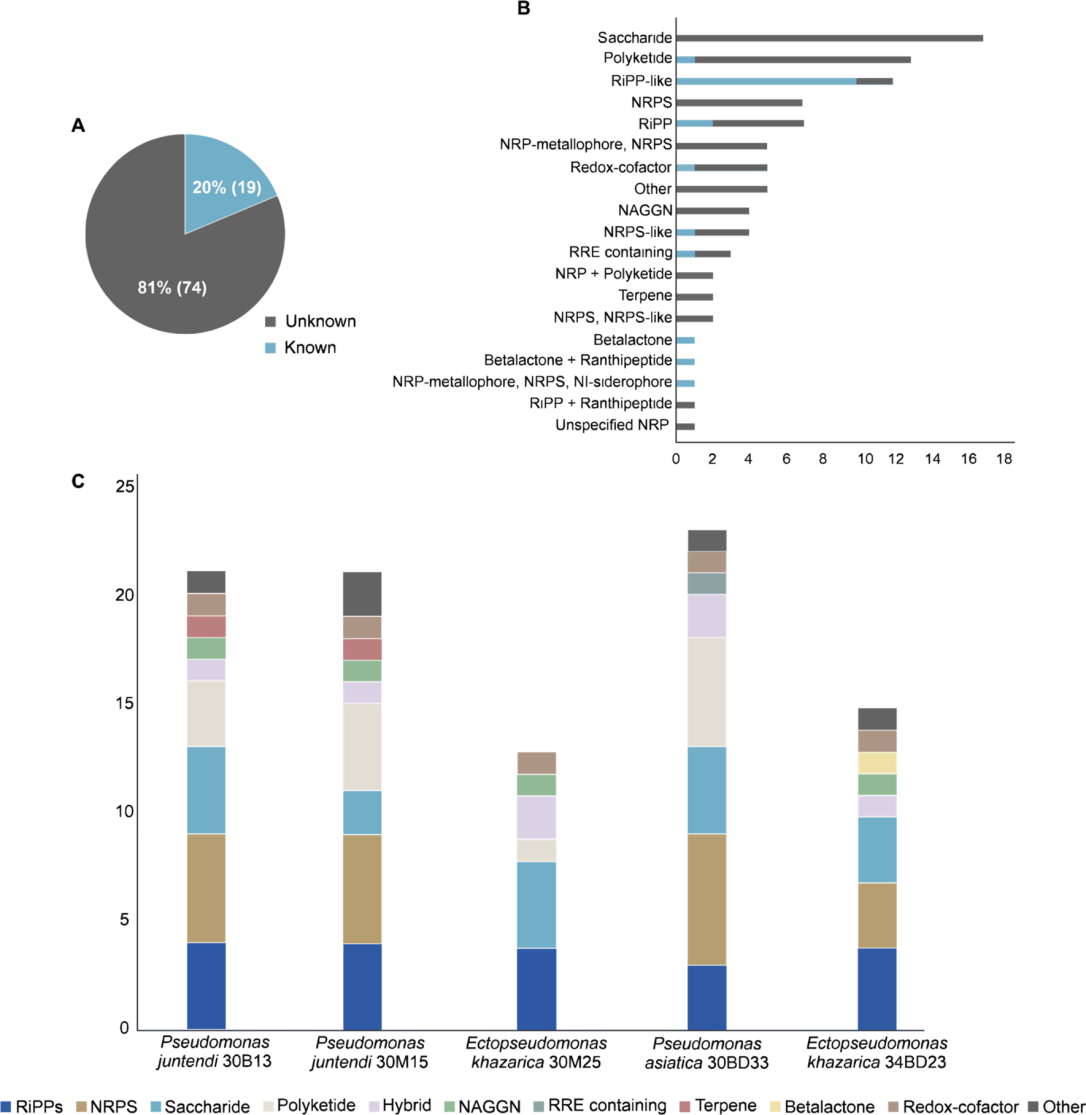
Total BGC diversity by categories across the five cave‐dwelling sponge‐associated Pseudomonads genomes. (A) Proportion of known and unknown BGCs. (B) Number of known and unknown BGCs of each detailed type. (C) BGC counts by categories distributed across the studied samples.

The antibacterial activity exhibited by the cave‐inhabiting sponge‐associated Pseudomonadaceae in this study suggests their potential to produce metabolites with antimicrobial properties. The in silico analysis of the five bacterial genomes revealed 93 BGCs of 19 compound classes (Figure 4; Table S6). Of the 93 BGCs, 74 (79.6%) had less than 80% gene cluster similarity with other known BGCs present in the MIBiG and/or antiSMASH database and were classified as unknown (Figure 4A). The most prevalent BGC types within the strains were saccharide, followed by polyketide, RiPP‐like, NRPS, among others in smaller numbers (Figure 4B). Overall, the number of BGCs per genome varied from 13 to 23 BGCs from 6 to 9 classes (Figure 4C). Notably, *P. asiatica* 30BD33 exhibited the highest BGC count, with 23 clusters identified, despite not presenting any exclusive BGC classes. Furthermore, only the *E. khazarica* strains 30M25 and 34BD23 contained betalactone‐encoding BGCs, while the *P. juntendi* strains 30M15 and 30B13 were found to produce terpenes. In general, the ARTS 2.0 analysis identified that 29 of the 93 (31.2%) identified BGCs in the Pseudomonadaceae strains were in proximity to a duplicated core gene or a known antibiotic‐resistance gene (Table S6).

### 3.5 Sequence‐Based Similarity Network of BGCs

To determine whether the observed biosynthetic potential was a species‐specific trait, a feature related to the cave‐dwelling sponge‐associated strains analysed in this study, or also linked to the isolation site of the strains, firstly, the overall similarity of the genomes was assessed and the BGC profiles were compared with those of reference strains from the same species available in the NCBI genome database (Table S3). Notably, ANI values > 98% were observed for *P. juntendi* and *E. hazarica*, whereas between *P. asiatica* strains a greater diversity was observed with ANI varying from 96.08% to 99.84% (Figures S3–S5).

**FIGURE 5:**
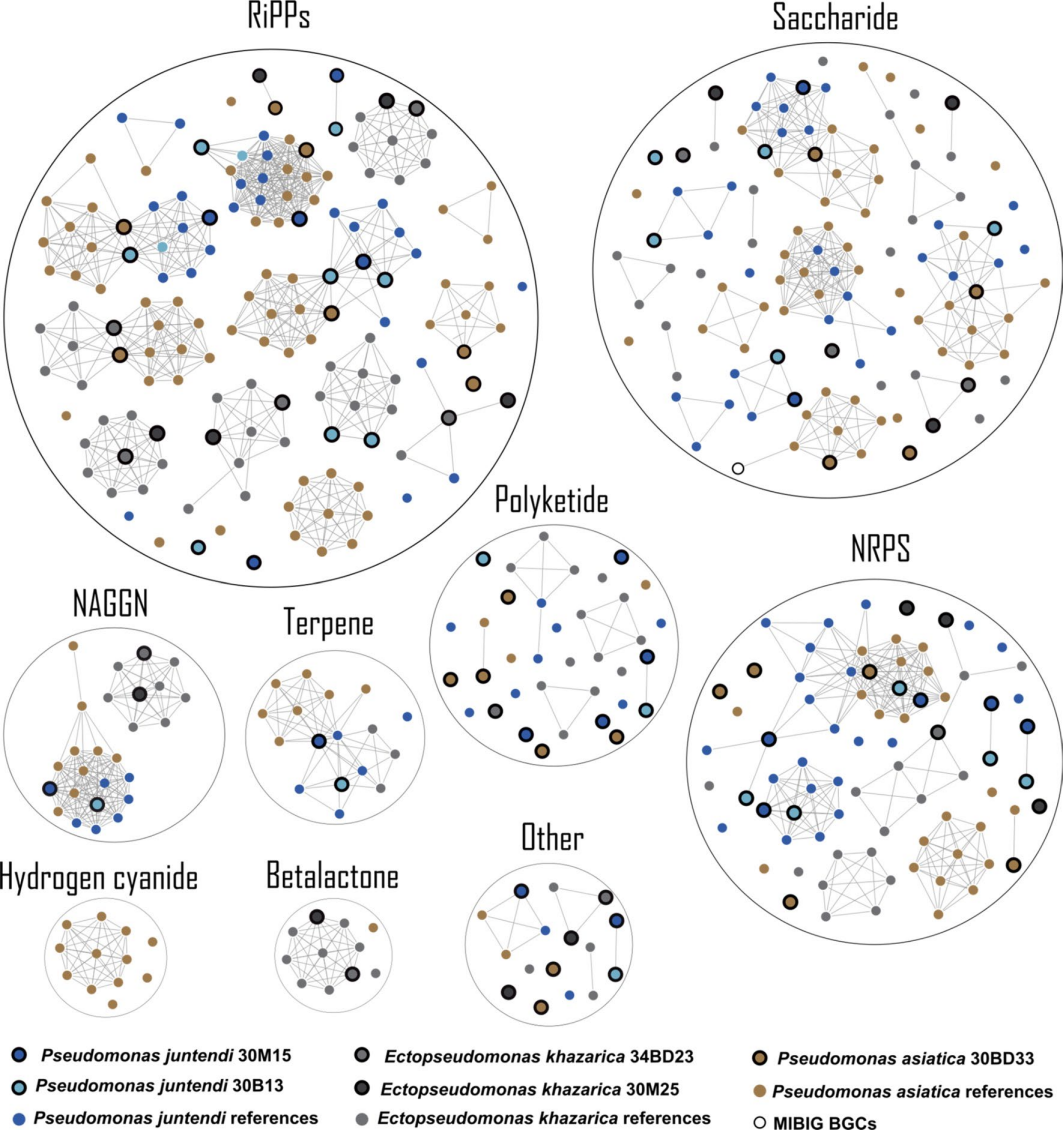
Sequence similarity network of 450 BGCs with a cutoff of 0.3 generated by BiG‐SCAPE v1.1.5. Each node represents an individual BGC, coloured according to the original sponge‐associated bacterial strains, and the similar BGCs were linked together as one cluster. The single‐node indicates a unique BGC in the network. NAGGN: N‐acetylglutaminylglutamine amide; RRE containing: RRE‐element containing cluster; NRPS: Non‐ribosomal peptide synthetase; RiPP: Ribosomally synthesised and post‐translationally modified peptides; Other: Cluster containing a secondary metabolite‐related protein that does not fit into any other category. BGCs of the sponge‐associated bacterial strains of the present study are highlighted with thick borders.

The analysis involved 450 BGCs and the sequence similarity network of the BGCs showed that the connected components corresponded to 159 GCFs of nine predominant types: ribosomally synthesised and post‐translationally modified peptides (RiPPs), nonribosomal peptide synthetases (NRPS), saccharide, polyketide, other, NAGGN, RRE containing, terpene and betalactone (Figure 5). Noteworthy, a total of 81 singletons were identified, and just one BGC family clustered with a MIBiG reference cluster.

Overall, it was possible to observe BGCs, particularly from RiPP, terpene, saccharide and NRPS, belonging to the same cluster family shared across the members of the three Pseudomoadaceae species. In contrast, the majority of them exhibited a tendency of clustering exclusively among isolates of the same species. This was especially true for *E. khazarica* strains, which exhibited only four cluster families containing BGCs shared with other species, whereas *E. asiatica* and *E. juntendi* displayed several BGCs in common with each other (Figure 5). Aside from those conserved in all species and those species‐specific, strain‐specific BGCs were also observed.

**FIGURE 6:**
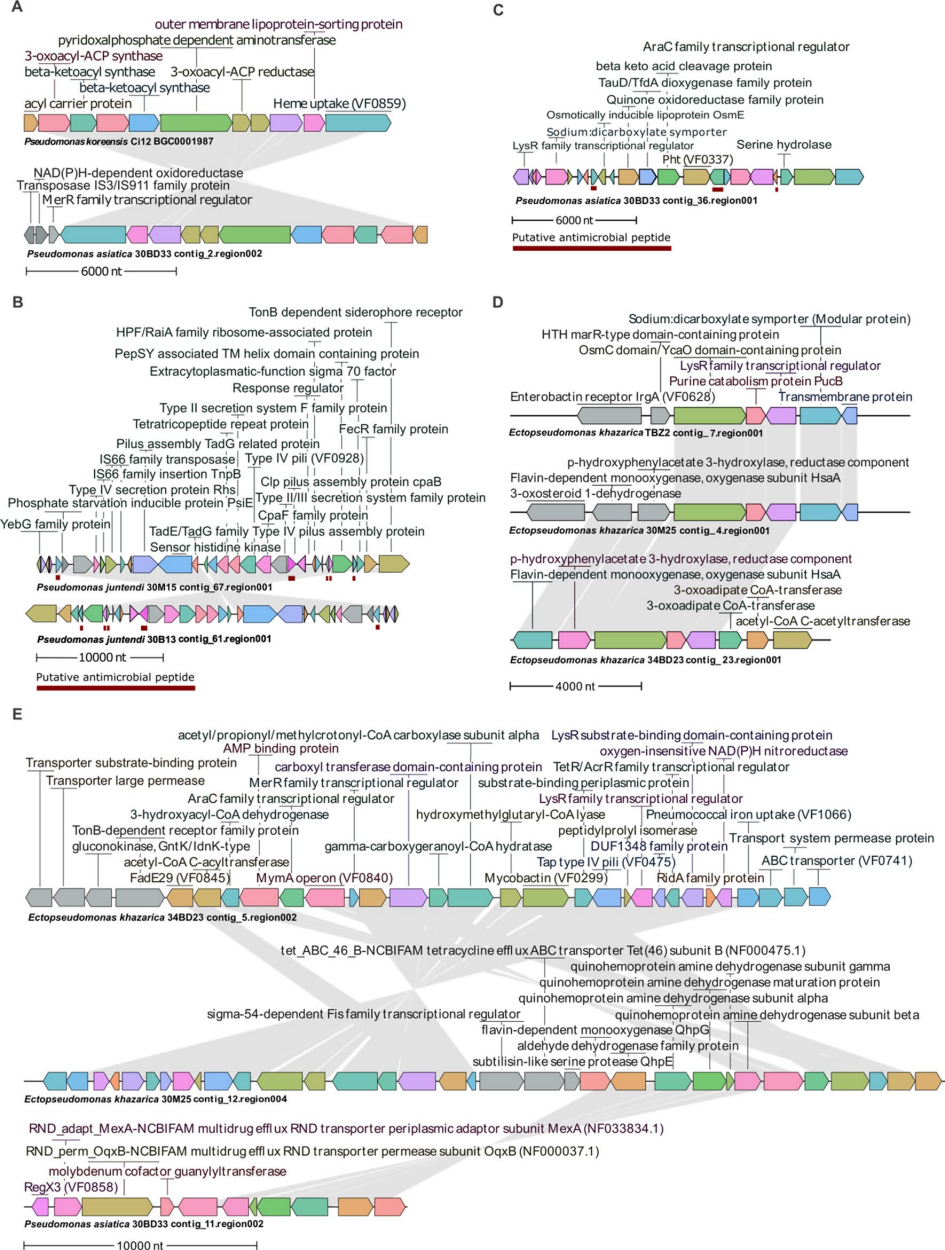
Most promising biosynthetic gene clusters (BGCs) putatively encoding antimicrobial molecules. (A) Koreenceine BGC synteny between the reference *Pseudomonas koreensis* strain C12 and *P. asiatica* 30BD33. (B) Unknown BGC found in *P. juntendi* strains 30M15 and 30B13 and (C) an unknown BGC identified exclusively in *P. asiatica* 30BD33. (D) RiPP‐like BGC present in *E. khazarica* reference strain TBZ2 and in 30M25 and 34BD23. (E) Hybrid ranthipeptide/betalactone BGC synteny between *E. khazarica* strains 34BD23 and 30M25, and in *P. asiatica* 30BD23. Putative antimicrobial peptide coding sequences predicted with AmpGram and AMP Scanner V2 are indicated by red lines. Identical colours represent the same CDS within the cluster.

More specifically, among the BGCs that presented high similarity (> 80%) with known clusters, koreenceine (Figure 6A) and icosalide (not shown) were identified in *P. asiatica* 30BD33 with 100% similarity. While the former grouped with other *P. asiatica* and with *Pseudomonas koreensis* strain CI12 present in the MIBiG database (Figure S6), the latter remained as a singleton. In addition, further investigation revealed another known BGC encoding for Bokeelamides shared between all *E. khazarica* strains. The cluster was complete in *E. khazarica* 30M25, and the main core biosynthetic genes exhibited > 98% identity and > 99% similarity by pairwise sequence alignment with *E. khazarica* EM133, from which this cluster was first identified and characterised (Campbell et al. 2024). In contrast, in *E. khazarica* 34BD23, it was lacking important genes and presented ~88% similarity and identity with this reference strain (Figure S7).

Additionally, as mentioned earlier, a great diversity of unknown BGCs was found. To further correlate this biosynthetic diversity with the antimicrobial activity observed in vitro for the strains here analysed, and to uncover potential new antimicrobial metabolites, each BGC of the sponge associated bacteria was further investigated considering: (1) completeness; (2) Transporters; (3) Resistance markers; (4) Regulators; (5) Biosynthetic genes; and/or (6) Putative antimicrobial peptides. Besides koreenceine (Figure 6A) and icosalide, which are validated antimicrobial compounds, the most promising antimicrobial candidate clusters are presented in Figure 6B–E. Firstly, among the *P. juntendi* specific BGCs, one family cluster comprising two related BGCs found in *P. juntendi* 30B13 and 30M15 sparked interest for being composed of secretion/assembly components, five small peptides predicted as AMPs with high score (0.8–1.0), a regulatory gene (FecR‐like), and nearby mobile elements (IS*66*/TnpB) (Figure 6B). SignalP 6.0 analysis of these AMP candidates indicated the presence of N‐terminal signals classified as ‘other’. Regarding those found in *P. asiatica* 30BD33, a singleton BGC presented three small peptides predicted as AMPs (scores 0.54–0.99), all SignalP‐positive, the first of which bears a lipoprotein signal. The locus also encodes regulatory proteins (LysR‐ and AraC‐like), a major facilitator superfamily (MFS) transporter, and several putative tailoring enzymes (Figure 6C). Finally, in *E. khazarica*, two unknown yet complete BGCs of types RiPP‐like and hybrid ranthipeptide/betalactone were observed (Figure 6D,E). The former was found in both *E. khazarica* 30M25 and 34BD23, and in TBZ2 (isolated from water samples) and contained a core biosynthetic gene encoding YcaO adjacent redox enzymes and a LysR‐family transcriptional (Figure 6D). However, a dedicated transporter for peptide export is not annotated within the cluster. On the other hand, the ranthipeptide/betalactone BGC found in all *E. khazarica* strains here analysed contains multiple AMP‐binding proteins, a Type II/IV secretion system component, and ABC transporter‐related proteins (Figure 6E; Figure S8).

## 4 Discussion

Sponge‐associated bacteria are recognised as key reservoirs of bioactive compounds in the marine environment, including potential antibacterial molecules against MDR bacteria (Devkar et al. 2023; Carroll et al. 2024). Moreover, previous studies conducted in submarine cave systems have highlighted the biotechnological relevance of microorganisms isolated from these habitats, reporting the production of diverse secondary metabolites with antimicrobial and other bioactive properties (Ghosh et al. 2017; Lo Giudice and Rizzo 2022; Kosznik‐Kwaśnicka et al. 2022). Notably, this study demonstrates that marine bacteria with anti‐ *Staphylococcus aureus* activity were isolated from 17 out of 18 (94%) cave‐dwelling sponges. Notably, studies on most of the sponge species analysed here are limited. With the exception of *Dysidea* spp. and *Xestospongia* spp. (Indraningrat et al. 2022; Zhang et al. 2022), this represents the first study focused on harnessing the antibacterial potential of the bacterial communities isolated from sponge species such as Axinyssa spp., *G. cavernicola* , *Dercitus (Stoeba) latex*, *Ectyoplasia ferox* , *Spirastrella hartmani* and *Topsentia ophiraphidites*.

In general, studies exploring the biotechnological potential of bacteria associated with cave‐dwelling sponges are scarce (Hentschel et al. 2001; Campbell et al. 2024). Canellas et al. (2024), for instance, were the first to demonstrate the antimicrobial potential of *Vibrio* spp. isolated from cave‐dwelling sponges of the class Calcarea from the Fernando de Noronha Archipelago in the Western Atlantic Ocean. Among 13 *Vibrio* spp. strains analysed, four were simultaneously active against *S. aureus* and *E. coli* (Campbell et al. 2024). Therefore, the present study expands the scope of previous research, pioneering the exploration of cave‐dwelling sponges from the Demospongiae class. Additionally, it reports bacteria with a broad spectrum of inhibition on indicator strains, including virulent and multidrug‐resistant pathogens highlighted by WHO's priority pathogens list (WHO 2024). This discovery underscores the untapped biotechnological potential of sponge‐associated bacteria and their promise as a source of antimicrobials to combat the rising threat of multidrug‐resistant pathogens.

In this study, strains belonging to the family Pseudomonadaceae stood out while being attributed to five out of the 10 strains that additionally showed strong activity against the resistant bacteria and were further selected for genome sequencing. When compared to reference Pseudomonadaceae genomes available in public databases, the general genome metrics of the five bioactive strains analysed in this study fall within the expected range for the family. Similar genome features have been described for environmental and clinical representatives of *Pseudomonas* and related genera, reflecting their versatile metabolic capacity and ecological plasticity (Palleroni 2015; Peix et al. 2018; Gomila et al. 2023).

Several Pseudomonads species are suitable for prospecting molecules with biotechnologically relevant properties as they are capable of producing a wide range of antimicrobials, biosurfactants and enzymes, especially those of marine origin (Canellas and Laport 2024). The bioactive sponge‐associated Pseudomonadaceae strains consistently exhibited clusters related to polyketide, RiPPs, NRPS, betalactone and ranthipeptide, all of which are reported to display antimicrobial activity and are represented by commercially approved antibiotics to treat different bacterial infections (Robinson et al. 2019; Chen et al. 2021; Ongpipattanakul et al. 2022). Additionally, ARTS 2.0 analysis identified that 29 of the 93 identified BGCs in the Pseudomonadaceae strains were in proximity to a duplicated core gene or a known antibiotic‐resistance gene. This genomic context indicate that these BGCs may encode for antibiotic compounds based on self‐resistant mechanisms of an antibiotic‐producing organism (Ziemert et al. 2016). Koreenceine, for instance, identified in *P. juntendi* 30M15, is a polyketide produced mainly by *Pseudomonas koreensis* that affects gene expression and exerts antibacterial activity against *Flavobacterium johnsoniae* (Hurley et al. 2022). Icosalide, on the other hand, is an unusual two‐tailed lipocyclopeptide antibiotic that was originally isolated from a fungal culture, but also reported in *Burkholderia gladioli* with activity against entomopathogenic bacteria (Dose et al. 2018).

Interestingly, a high proportion of the observed BGCs exhibited low similarity (< 80%) to known clusters, and their functions could not be ascertained. Therefore, a comparative genomics analysis, based on publicly available genomes, was performed to assess the extent to which the biosynthetic potential of the marine sponge‐derived strains identified in this study is shared with related strains isolated from other ecological contexts and geographical regions. It is worth noting that, even though efforts were made to include genomes from different ecological origins whenever possible, this was not always achievable. For example, most *E. khazarica* strains available in the NCBI database (as of early October 2025) and consequently analysed here were isolated from aquatic environments, whereas those from *P. juntendi* were mainly isolated from human patients. This was further supported by OrthoANI analysis, which revealed greater diversity among *P. asiatica* strains, which is consistent with their varied sources of isolation, including human, soil, animal, and water. Still, the comparative studies provided insights into the distribution and possible role of the uncharacterized BGCs. For instance, the analysis revealed a cluster encoding for bokeelamides, which are recently described lipopeptides derived from moon snail egg mass‐associated *E. khazarica* strain EM133 (Campbell et al. 2024). Initial hints indicate that this molecule could be acting as siderophores; however, no antibacterial activity against *S. aureus* and *E. coli* , antifungal, antibiofilm, or red blood cell lysis was observed at concentrations up to 50 μM (Campbell et al. 2024). Our analysis further revealed that the BGC underlying bokeelamide biosynthesis is present in all analysed *E. khazarica* strains, albeit with varying degrees of completeness. This widespread occurrence suggests a potentially important role for this molecule within the species, particularly in strains from marine environments.

Regarding the remaining unknown BGCs, given the paucity of experimentally validated data in vitro, their potential products and biological functions remain largely speculative. Still, functional predictions related to antimicrobial molecules were inferred based on genomic context, including neighbouring genes, conserved domains, and synteny, providing a foundation for prioritising BGCs in future experimental studies. In this sense, four BGCs across the three Pseudomonadaceae species were particularly notable. Remarkably, within *E. khazarica*, a core biosynthetic gene encoding YcaO, a well‐characterised cyclodehydratase involved in the formation of heterocyclic rings such as thiazolines and oxazolines (Zheng and Nair 2023), was identified in a RiPP‐like BGC present in strains 30M25, 34BD23 and TBZ2. This highlights the potential of these clusters to produce modified peptides with diverse bioactivities, complementing the ranthipeptide/betalactone BGCs containing ABC transporters that may function as immunity genes.

Among the vast repertoire of molecules with antibacterial activity, small peptides stand out as a particularly promising avenue, acting either by disrupting negatively charged bacterial membranes or through intracellular targets (Zhang et al. 2021). These AMPs exhibit efficacy even against drug‐resistant bacteria, making them compelling candidates to further investigate the antimicrobial activity observed in this study. Consistently, by complementing tools such as AMP Scanner V2 and AmpGram, eight AMPs harbouring peptide signals were found within two BGCs found in *P. juntendi* 30M15 and 30B13, and *P. asiatica* 30BD33. Interestingly, both clusters also harboured genes that may participate in peptide regulation, post‐translational processing, and transport.

Overall, the present study provides a comprehensive characterisation of the biosynthetic potential of sponge‐associated Pseudomonadaceae strains endowed with antimicrobial activity. In addition, it presents a particular focus on *E. khazaria*, *P. asiatica* and *P. juntendi*, species that were only recently described (Tarhriz et al. 2020; Tohya, Watanabe, Teramoto, Uechi, et al. 2019; Tohya, Watanabe, Teramoto, Shimojima, et al. 2019). By elucidating promising BGCs, including putative AMP candidates, it establishes data for future functional genomics research. The characterisation of these novel clusters will enrich public databases, bolster genome mining efforts, and pave the way for discovering new bioactive compounds active against drug‐resistant pathogens, offering new avenues for managing bacterial infections.

## Author Contributions

**Gabriel Rodrigues Dias:** writing – original draft, writing – review and editing, data curation, investigation, conceptualization, methodology, formal analyses, validation, visualization. **Bruno Francesco Rodrigues de Oliveira:** writing – review and editing, conceptualization, data curation, methodology, validation, supervision. **Joana Sandes:** writing – review and editing, methodology, investigation. **Guilherme Muricy:** writing – review and editing, resources, funding acquisition. **Marinella Silva Laport:** conceptualization, funding acquisition, supervision, project administration, writing – review and editing, resources.

## Funding

This work was supported by Fundação Carlos Chagas Filho de Amparo à Pesquisa do Estado do Rio de Janeiro, E‐26/211.284/2021, E‐26/204.045/2024, E‐26/204.575/2024. Conselho Nacional de Desenvolvimento Científico e Tecnológico, 443302/2019‐7, 309158/2023‐0, 405020/2023‐6, 441722/2024‐5. Coordenação de Aperfeiçoamento de Pessoal de Nível Superior, 001.88887.959778/2024‐00. Office of Naval Research, N62909‐23‐1‐2021.

## Conflicts of Interest

The authors declare no conflicts of interest.

## Supporting information


**Figure S1:** Identification by MALDI‐TOF MS of the bioactive sponge‐associated bacterial strains.
**Figure S2:** Cluster analysis of band patterns of Pseudomonadaceae strains isolated from marine sponges obtained by BOX‐PCR. The similarity percentage is identified in the dendrogram derived from the unweighted pair group method using arithmetic means and based on the DICE coefficient. Strains are separated by two colours to indicate the sponge sample from which they were isolated.
**Figure S3:** Average nucleotide identity (ANI) of *P. juntendi* strains determined by OrthoANI. Strains isolated in the present study are highlighted in bold.
**Figure S4:** Average nucleotide identity (ANI) of *E. khazarica* strains determined by OrthoANI. Strains isolated in the present study are highlighted in bold.
**Figure S5:** Average nucleotide identity (ANI) of *P. asiatica* strains determined by OrthoANI. Strains isolated in the present study are highlighted in bold.
**Figure S6:** Koreenceine gene cluster family identified across *Pseudomonas asiatica* strains using BiG‐SCAPE v1.1.5. The *Pseudomonas koreensis* C12 BGC was retrieved from the MIBiG v3.0 database and used as a reference. Identical colours represent the same CDS within the cluster.
**Figure S7:** BGCs encoding Bokeelamides identified across *Ectopseudomonas khazarica* strains. (A) Synteny of the genetic cluster between *E. khazarica* strains 30M25 and 34BD23 of the present study and the reference strain *E. khazarica* EM133. (B) Gene cluster families of Bokeelamides identified across *E. khazarica* strains, as determined using BiG‐SCAPE v1.1.5. Note that for *E. khazarica* strain BC_CKDN230030182‐1A_HGKHYDSX7 the region appears to be split into two separate BGCs. Identical colours represent the same CDS within the cluster.
**Figure S8:** Gene cluster family of an unknown hybrid ranthipeptide/betalactone BGC identified across *Ectopseudomonas khazarica* and *Pseudomonas asiatica* strains using BiG‐SCAPE v1.1.5. Identical colours represent the same CDS within the cluster.
**Table S1:** Summary of the main characteristics of the sponge samples of this study.
**Table S2:** Bacterial strains used as indicators in the secondary screening stage with their origin, antimicrobial resistance profiles and other relevant features.
**Table S3:** Whole genome‐based taxonomic analysis performed with GTDB‐Tk of the five bioactive sponge‐associated Pseudomonadaceae strains.
**Table S4:** Pseudomonadaceae strains included as reference controls in the BGCs mining approach.
**Table S5:** Genome metrics of the five bioactive sponge‐associated Pseudomonadaceae strains.
**Table S6:** Diversity of BGCs across the five bioactive sponge‐associated Pseudomonadaceae strains.

## Data Availability

The data that supports the findings of this study are available in the Supporting Information of this article.
