# Supplementary material for: Gems From the Caves: Genomic Insights Into the Biosynthetic Potential of Antimicrobial‐Producing Bacteria Associated With Tropical Cave‐Dwelling Sponges
Source: Environ Microbiol. 2026 Jan 21;28(1):e70244. doi: 10.1111/1462-2920.70244 (PMC12823289; doi:10.1111/1462-2920.70244)
Supplement: Supplementary file 1 — Figure S1: Identification by MALDI‐TOF MS of the bioactive sponge‐associated bacterial strains. Figure S2: Cluster analysis of band patterns of Pseudomonadaceae strains isolated from marine sponges obtained by BOX‐PCR. The similarity percentage is identified in the dendrogram derived from the unweighted pair group method using arithmetic means and based on the DICE coefficient. Strains are separated by two colours to indicate the sponge sample from which they were isolated. Figure S3: Average nucleotide identity (ANI) of P. juntendi strains determined by OrthoANI. Strains isolated in the present study are highlighted in bold. Figure S4: Average nucleotide identity (ANI) of E. khazarica strains determined by OrthoANI. Strains isolated in the present study are highlighted in bold. Figure S5: Average nucleotide identity (ANI) of P. asiatica strains determined by OrthoANI. Strains isolated in the present study are highlighted in bold. Figure S6: Koreenceine gene cluster family identified across Pseudomonas asiatica strains using BiG‐SCAPE v1.1.5. The Pseudomonas koreensis C12 BGC was retrieved from the MIBiG v3.0 database and used as a reference. Identical colours represent the same CDS within the cluster. Figure S7: BGCs encoding Bokeelamides identified across Ectopseudomonas khazarica strains. (A) Synteny of the genetic cluster between E. khazarica strains 30M25 and 34BD23 of the present study and the reference strain E. khazarica EM133. (B) Gene cluster families of Bokeelamides identified across E. khazarica strains, as determined using BiG‐SCAPE v1.1.5. Note that for E. khazarica strain BC_CKDN230030182‐1A_HGKHYDSX7 the region appears to be split into two separate BGCs. Identical colours represent the same CDS within the cluster. Figure S8: Gene cluster family of an unknown hybrid ranthipeptide/betalactone BGC identified across Ectopseudomonas khazarica and Pseudomonas asiatica strains using BiG‐SCAPE v1.1.5. Identical colours represent the same CDS within the clust [file EMI-28-e70244-s001.docx]

***Supplementary files***

**Gems from the caves: genomic insights into the biosynthetic potential of antimicrobial-producing bacteria associated with tropical cave-dwelling sponges**

Gabriel Rodrigues Dias^1^, Bruno Francesco Rodrigues de Oliveira^2^, Joana Sandes^3^, Guilherme Muricy^3^, Marinella Silva Laport^1*^

^1^Instituto de Microbiologia Paulo de Góes, Universidade Federal do Rio de Janeiro, Av. Carlos Chagas Filho, 373, Cidade Universitária 21941-902, Rio de Janeiro, Brazil

^2^Departamento de Microbiologia e Parasitologia, Instituto Biomédico, Universidade Federal Fluminense, 24210-130 Niterói, RJ, Brazil

^3^Departamento de Invertebrados, Museu Nacional, Universidade Federal do Rio de Janeiro. Quinta da Boa Vista, s/nº, São Cristóvão. 20940–040 Rio de Janeiro, RJ, Brazil

***ORCiD of the authors:*** 0000-0003-1189-3900 (GRD), 0000-0001-6029-3608 (BFRO), 0000-0003-0311-8250 (JS), 0000-0002-1705-3673 (GM), 0000-0001-5252-0671 (MSL)

***Corresponding author:** marinella@micro.ufrj.br (M.S. Laport*)

**
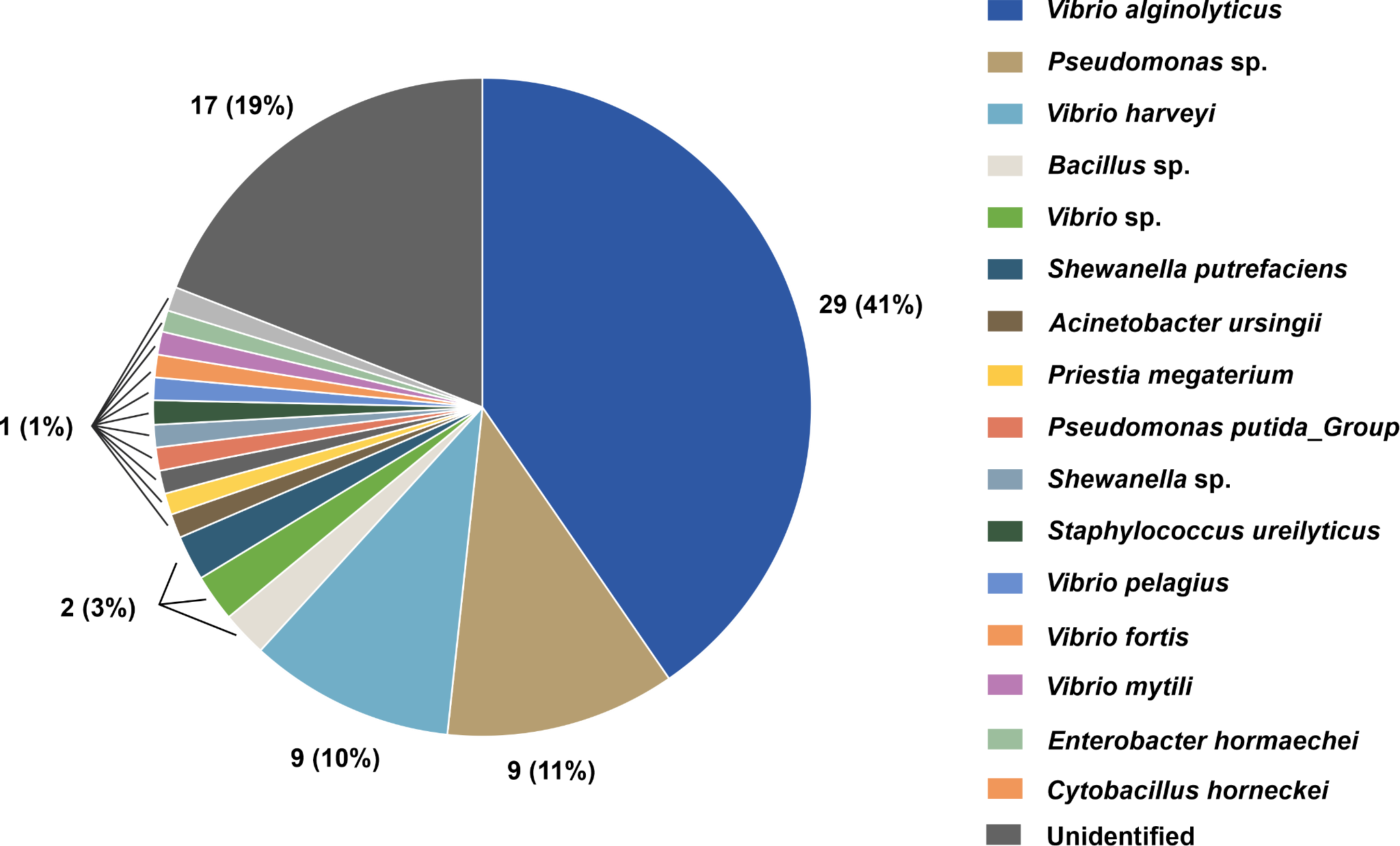
**

**Figure S1.** Identification by MALDI-TOF MS of the bioactive sponge-associated bacterial strains.

**
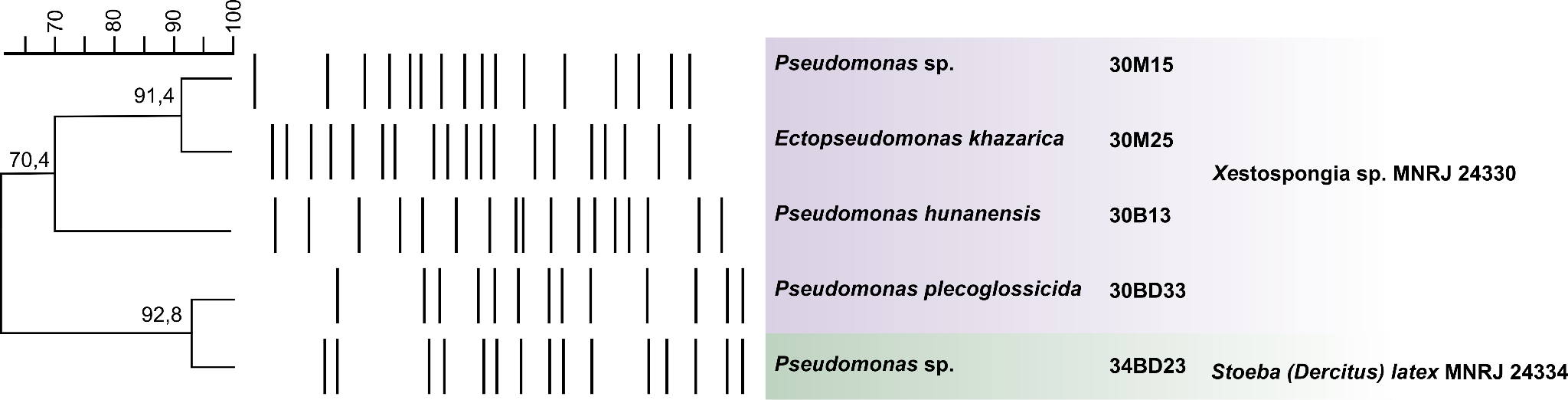
**

**Figure S2.** Cluster analysis of band patterns of Pseudomonadaceae strains isolated from marine sponges obtained by BOX-PCR. The similarity percentage is identified in the dendrogram derived from the unweighted pair group method using arithmetic means and based on the DICE coefficient. Strains are separated by two colors to indicate the sponge sample from which they were isolated.

**
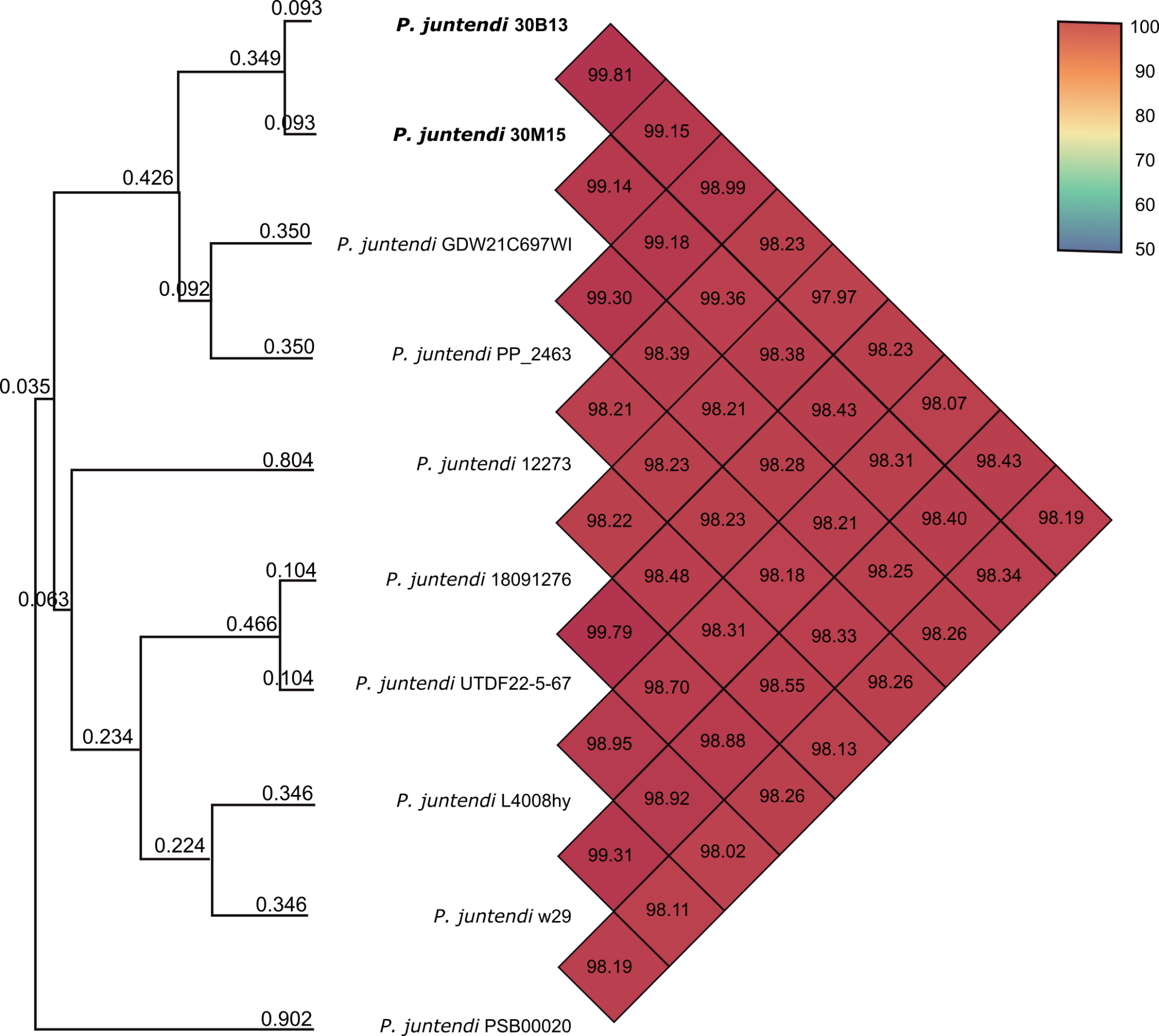
**

**Figure S3.** Average nucleotide identity (ANI) of *P. juntendi* strains determined by OrthoANI. Strains isolated in the present study are highlighted in bold.

**
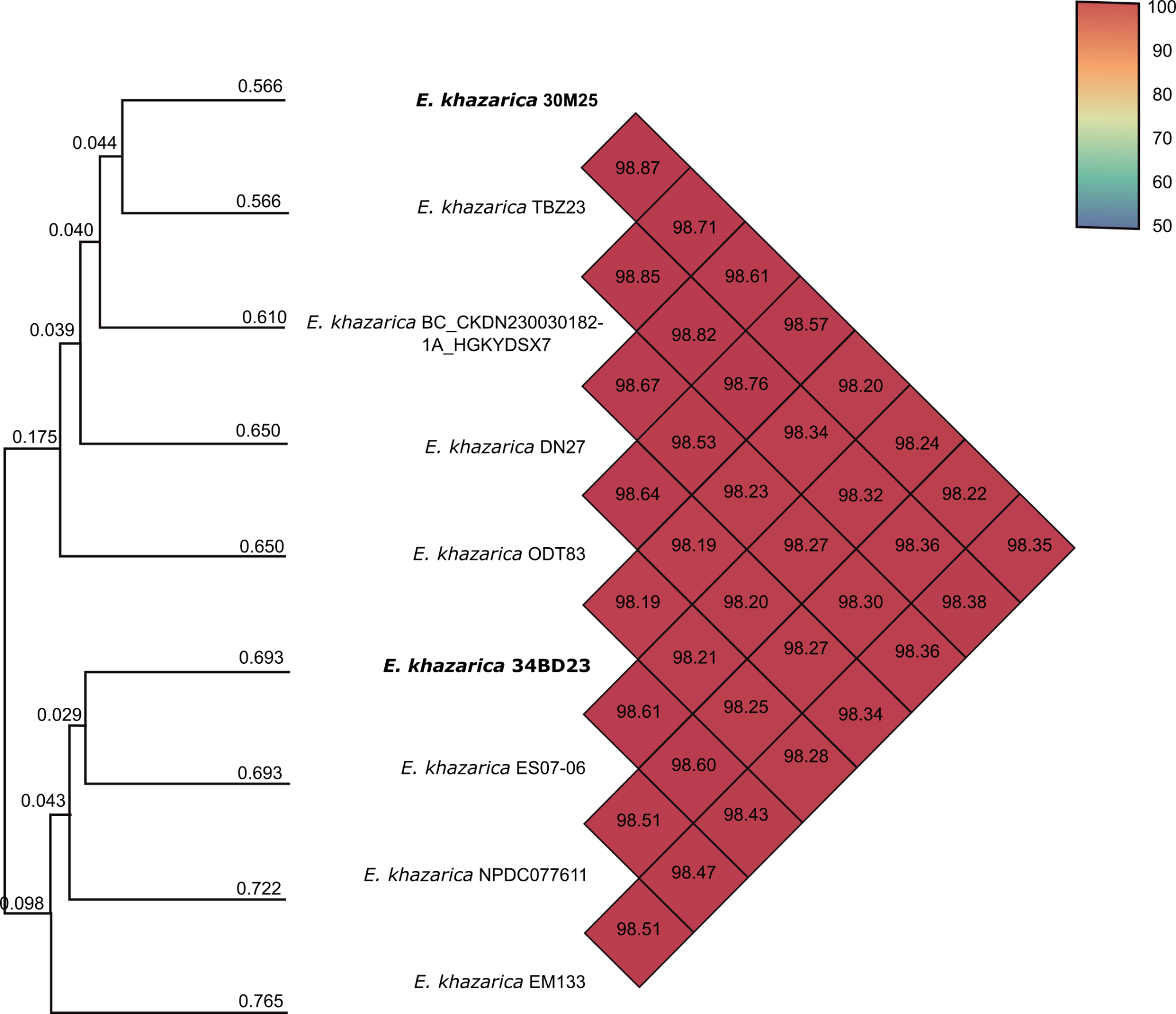
**

**Figure S4.** Average nucleotide identity (ANI) of *E. khazarica* strains determined by OrthoANI. Strains isolated in the present study are highlighted in bold.

**
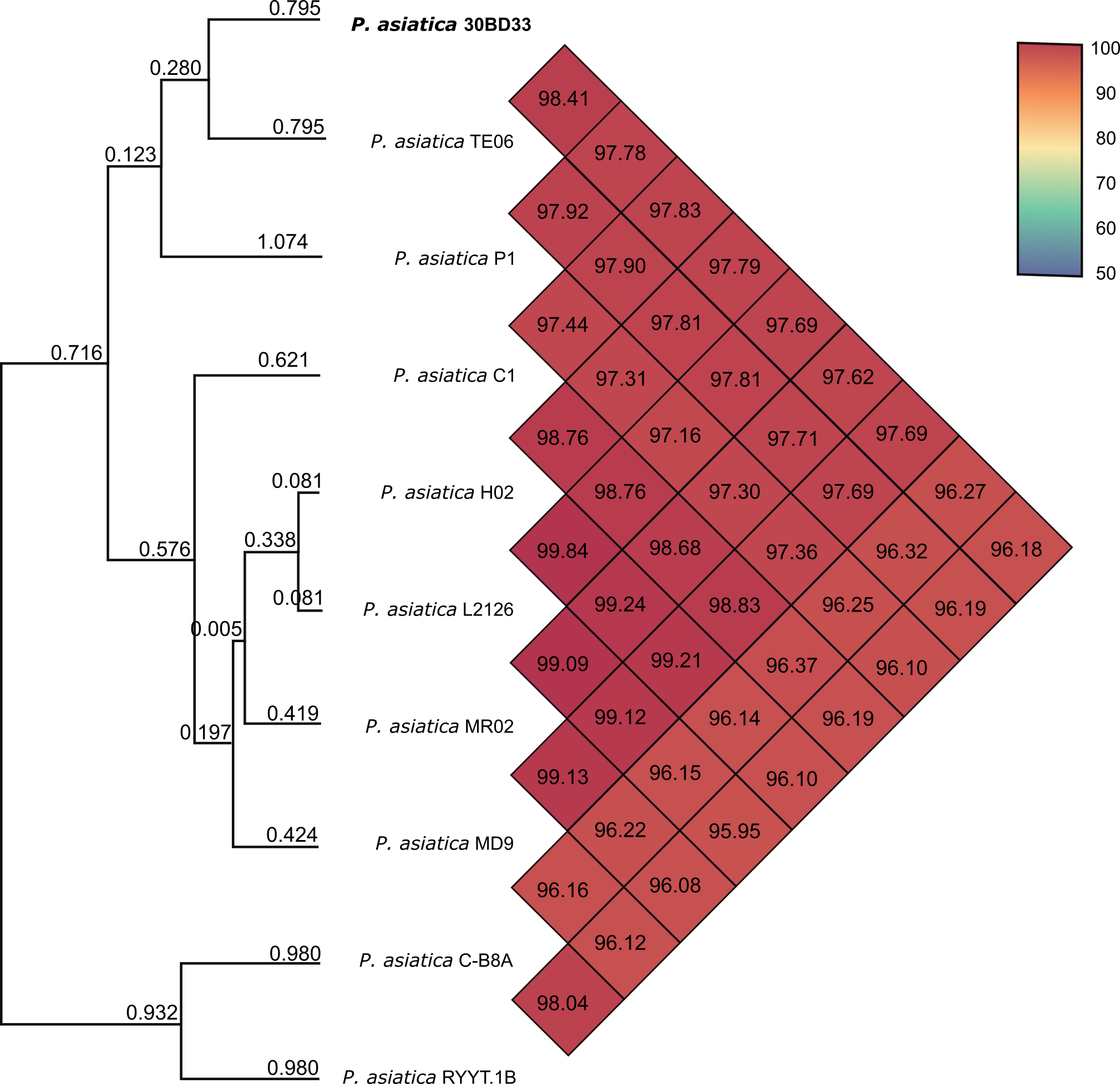
**

**Figure S5.** Average nucleotide identity (ANI) of *P. asiatica* strains determined by OrthoANI. Strains isolated in the present study are highlighted in bold.


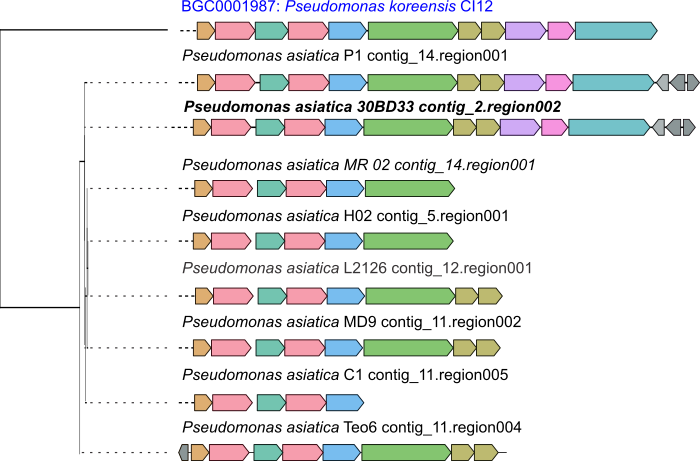


**Figure S6.** Koreenceine gene cluster family identified across *Pseudomonas asiatica* strains using BiG-SCAPE v1.1.5. The *Pseudomonas koreensis* C12 BGC was retrieved from the MIBiG v3.0 database and used as a reference. Identical colors represent the same CDS within the cluster.


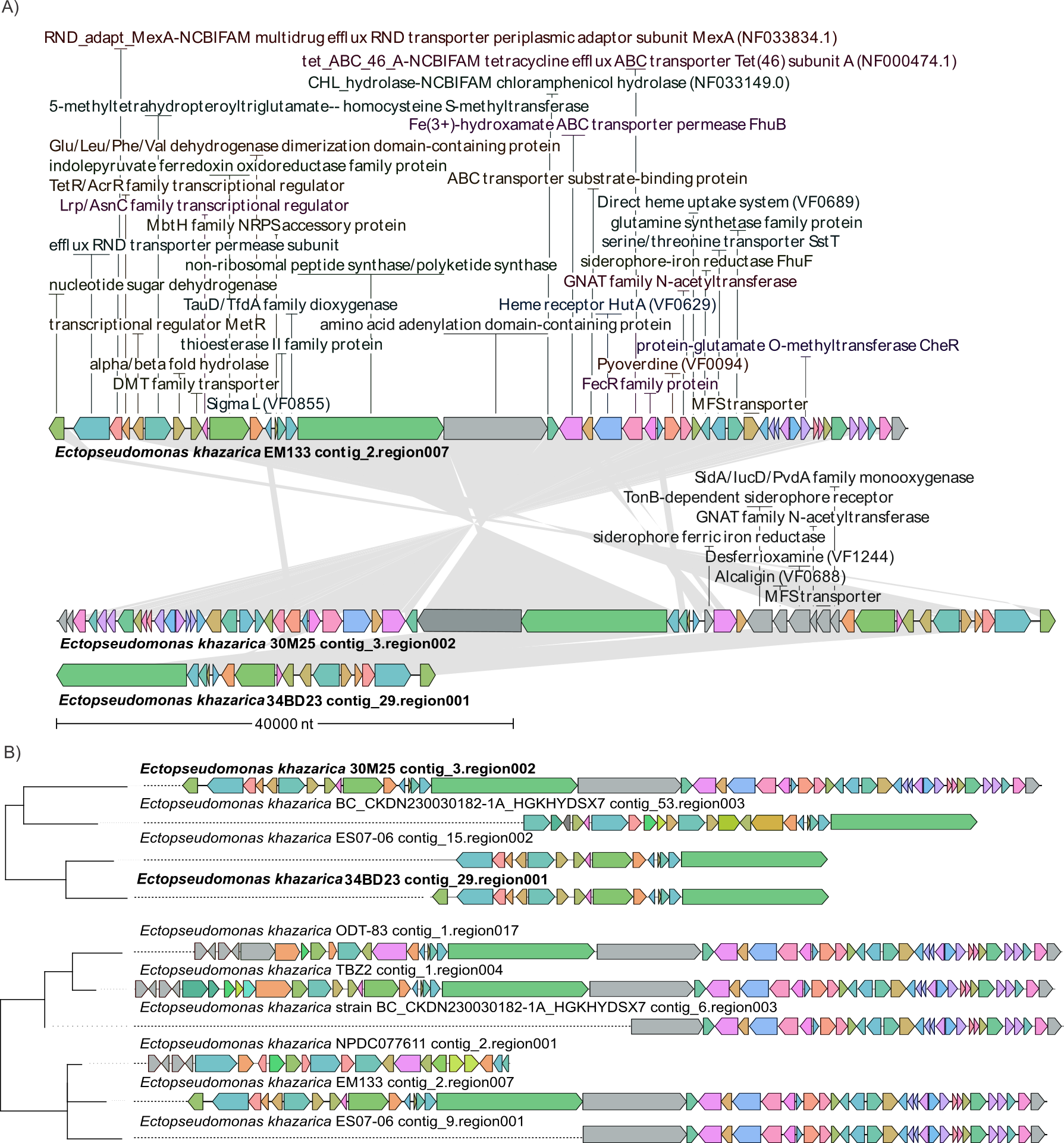


**Figure S7.** BGCs encoding Bokeelamides identified across *Ectopseudomonas khazarica* strains. A) Synteny of the genetic cluster between *E. khazarica* strains 30M25 and 34BD23 of the present study and the reference strain *E. khazarica* EM133. B) Gene cluster families of Bokeelamides identified across *E. khazarica* strains, as determined using BiG-SCAPE v1.1.5. Note that for *E. khazarica* strain BC_CKDN230030182-1A_HGKHYDSX7 the region appears to be split into two separate BGCs. Identical colors represent the same CDS within the cluster.


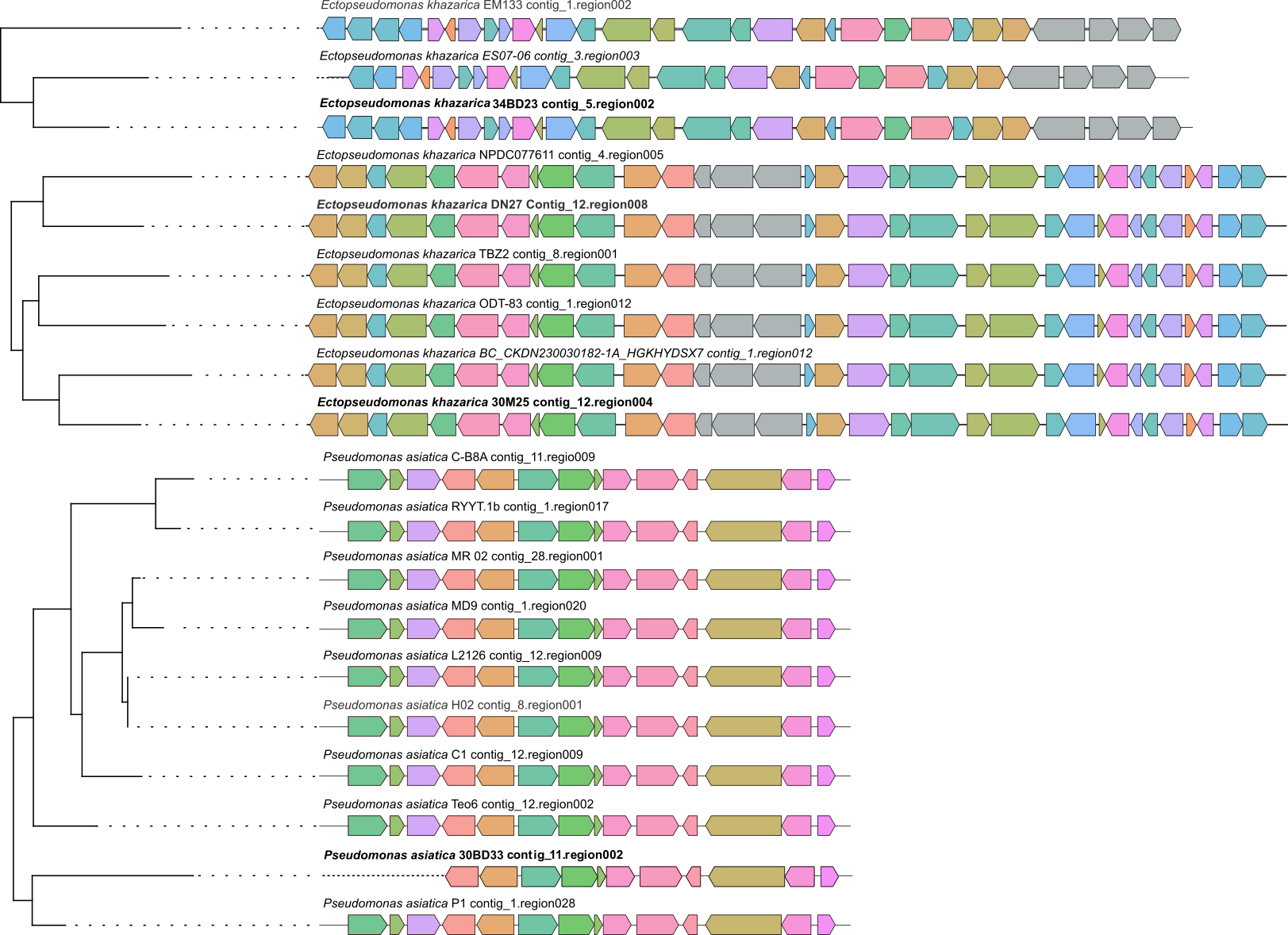


**Figure S8.** Gene cluster family of an unknown hybrid ranthipeptide/betalactone BGC identified across *Ectopseudomonas khazarica* and *Pseudomonas asiatica* strains using BiG-SCAPE v1.1.5. Identical colors represent the same CDS within the cluster.

**Table S1.** Summary of the main characteristics of the sponge samples of this study

| **Voucher** | **Species** | **Collection site** | **Cave zones** | **Depth (m)** | **Morphological observations** |
| --- | --- | --- | --- | --- | --- |
| MNRJ 24270 | *Axinyssa* sp. 1 | Sapata cave | Semi-dark zone | 11.3-16.5 | Cushion-shaped, cream to orange color and ridges or conules at the surface |
| MNRJ 29271 | *Axinyssa* sp. 2 | Sapata cave | Semi-dark zone | 11.3-16.5 | Thick encrusting shape, cream color and smooth surface. |
| MNRJ 24334 | *Dercitus (Stoeba) latex* | Ilha do Meio cave | Semi-dark zone | 6.9-10.0 | Reddish-brown color, thick encrusting to massive shape |
| MNRJ 29333 | *Dercitus (Stoeba) latex* | Ilha do Meio cave | Semi-dark zone | 6.9-10.0 |  |
| MNRJ 29278 | *Dysidea* sp. | Sapata cave | Semi-dark zone | 11.3-16.5 | Thick encrusting shape, cream color , with conulose and translucid surface |
| MNRJ 29250 | *Ectyoplasia ferox* | Sapata cave | Semi-dark zone | 11.3-16.5 | Orange-yellow to brownish red color, thick encrusting to massive shape |
| MNRJ 29273 | *Ectyoplasia ferox* | Sapata cave | Semi-dark zone | 11.3-16.5 |  |
| MNRJ 24056 | *Gastrophanella cavernicola* | Sapata cave | Transition zone | 11.3-16.5 | Cream color, massive-lobate shape |
| MNRJ 24271 | *Gastrophanella cavernicola* | Ilha do Meio cave | Transition zone | 6.9-10.0 |  |
| MNRJ 24350 | *Gastrophanella cavernicola* | Ilha do Meio cave | Transition zone | 6.9-10.0 |  |
| MNRJ 29245 | *Gastrophanella cavernicola* | Sapata cave | Transition zone | 11.3-16.5 |  |
| MNRJ 29247 | *Gastrophanella cavernicola* | Sapata cave | Transition zone | 11.3-16.5 |  |
| MNRJ 29263 | *Spirastrella hartmani* | Ilha do Meio cave | Semi-dark zone | 6.9-10.0 | Orange to slamon color, thinly encrusting shape |
| MNRJ 24317 | *Topsentia ophiraphidites* | Sapata cave | Semi-dark zone | 11.3-16.5 | White color, irregular massive shape, with digitiform projection |
| MNRJ 29269 | *Topsentia ophiraphidites* | Sapata cave | Semi-dark zone | 11.3-16.5 |  |
| MNRJ 24330 | *Xestospongia* sp. | Ilha do Meio cave | Cave transition zone | 6.9-10.0 | White to pink color, thinly encrusting shape, with mucous |
| MNRJ 29321 | *Xestospongia* sp. | Ilha do Meio cave | Cave transition zone | 6.9-10.0 |  |
| UFRJPOR 9129 | *Xestospongia* sp. | Ilha do Meio cave | Semi-dark zone | 6.9-10.0 |  |

**Table S2.** Bacterial strains used as indicators in the secondary screening stage with their origin, antimicrobial resistance profiles and other relevant features.

| **Indicator strains** | **Antimicrobial resistance profile and other relevant characteristics** | **Sample source** |
| --- | --- | --- |
| *Acinetobacter baumannii* | Resistant to oxacillin | Human blood |
| *Aeromonas* sp_1_. | Resistant to colistin and carbapenem | Water |
| *Aeromonas* sp_2_. | Resistant to colistin and beta-lactams and mercury | Water |
| *Citrobacter freundii* | Resistant to aminoglycosides, beta-lactams, macrolides and quinolones (MDR) | Water |
| *Enterococcus faecalis* | Resistant to streptomycin | Human feces |
| *Enterobacter* sp. | Resistant to quinolones | Human |
| *Escherichia coli* | Resistant to quinolones | Human |
| *Staphylococcus epidermidis* | Resistant to beta-lactams, quinolones and tetracyclines (MDR) | Human blood |
| *Staphylococcus epidermidis* ATCC 35984 | Biofilm producer | Human blood |
| *Klebsiella pneumoniae* | Resistant to beta-lactams, fluoroquinolones and aminoglycosides (MDR) | Human blood |

MDR: multidrug-resistant bacteria; ATCC: American Type Culture Collection.

**Table S3.** Whole genome-based taxonomic analysis performed with GTDB-Tk of the five bioactive sponge-associated Pseudomonadaceae strains.

| **Host sponge** | **Bacterial strains** | **Closest genome reference** | **Closest genome taxonomy** | **ANI (%)** | **GenBank accession number** |
| --- | --- | --- | --- | --- | --- |
| MNRJ 24330  *Xestospongia* sp. | 30B13 | GCF_009932375.1 | *Pseudomonas juntendi* | 97.7 | GCF_049166885.1 |
|  | 30M15 | GCF_009932375.1 | *Pseudomonas juntendi* | 97.93 | GCF_049166845.1 |
|  | 30M25 | GCF_009932335.1 | *Ectopseudomonas khazarica* | 97.78 | GCF_049166815.1 |
|  | 30BD33 | GCF_004521985.1 | *Pseudomonas asiatica* | 98.70 | GCF_049166835.1 |
| MNRJ 24334 *Dercitus (Stoeba) latex* | 34BD23 | GCF_004521985.1 | *Ectopseudomonas khazarica* | 98.21 | GCF_049166775.1 |

**Table S4.** Pseudomonadaceae strains included as reference controls in the BGCs mining approach.

| **Species** | **Strain** | **Isolation source** | **GenBank accession number** |
| --- | --- | --- | --- |
| *Ectopseudomonas khazarica* | ODT-83 | Oyster  (*Crassostrea gigas* - China) | GCA_017915135.1 |
|  | TBZ2 | Water sample (England) | GCA_004521985.1 |
|  | EM133 | Moon snail (USA) | GCA_043965275.1 |
|  | BC_CKDN230030182-1A_HGKHYDSX7 | Shrimp (Ecuador) | GCF_044540705.1 |
|  | DN27 | Biofilm (China) | GCF_036861155.1 |
|  | ES07-06 | Marine Biofilm (metagenomic assembly - China) | GCF_048265825.1 |
|  | NPDC077611 | Non-specified | GCF_044542835.1 |
| *Pseudomonas asiatica* | Teo6 | Activated sludge (Germany) | GCF_034358925.1 |
|  | P1 | Sediment of a drainage channel (China) | GCF_028752155.1 |
|  | H02 | Human urine (Spain) | GCF_041353515.1 |
|  | RYYT.1b | Soil (China) | GCF_030291675.1 |
|  | MD9 | Poultry farm (China) | GCF_024494485.1 |
|  | MR 02 | River water (India) | GCF_002797475.1 |
|  | L2126 | Human stool (China) | GCF_049561255.1 |
|  | C-B8A | Soil, vegetable field (China) | GCF_040214835.1 |
|  | C1 | Waste Water Treatment Plant (South Korea) | GCF_014656565.1 |
| *Pseudomonas juntendi* | L4008hy | Human feces (China) | GCA_037094715.1 |
|  | w29 | Wastewater influent (USA) | GCF_051905765.1 |
|  | 12273 | Human urine (Brazil) | GCF_014062265.1 |
|  | PP_2463 | Human urine (China) | GCF_021560075.1 |
|  | GDW21C697WI | Chicken (China) | GCA_028994055.1 |
|  | UTDF22-5-67 | Soil (USA) | GCF_040750175.1 |
|  | PSB00020 | Human sputum (USA) | GCF_016009085.1 |
|  | 18091276 | Human (metagenomic assembly - China) | GCF_021725455.1 |

**Table S5.** Genome metrics of the five bioactive sponge-associated Pseudomonadaceae strains.

| **Genome features** | **Bacterial strains** | | | | |
| --- | --- | --- | --- | --- | --- |
|  | **30B13** | **30M15** | **30M25** | **30BD33** | **34BD23** |
| **Completeness (%)** | 99.98 | 99.98 | 100.0 | 100.0 | 100.0 |
| **Contamination (%)** | 0.59 | 1.55 | 0.0 | 0.21 | 0.01 |
| **Contigs** | 222 | 242 | 64 | 318 | 72 |
| **N50 (contigs - kb)** | 41.209 | 42.726 | 169.751 | 34.381 | 122.751 |
| **Genome Size (Mpb)** | 5.45 | 5.68 | 5.46 | 5.92 | 5.29 |
| **Coverage** | 195x | 211x | 197x | 195x | 205x |
| **GC content (%)** | 62.2 | 62.3 | 64.7 | 62.7 | 64.6 |
| **rRNA** | 6 | 4 | 3 | 6 | 3 |
| **tRNA** | 52 | 50 | 47 | 50 | 50 |
| **CDS** | 5403 | 4997 | 5433 | 5119 | 4907 |
| **Coding density (%)** | 88.4 | 88.5 | 89.5 | 87.9 | 89.5 |
| **CDS with COG** | 4977 | 4761 | 4950 | 5122 | 4723 |
| **CRISPR** | 2 | 1 | 3 | 5 | 1 |
| **Cas proteins** | 10 | 10 | 1 | 10 | 1 |

**Table S6.** Diversity of BGCs across the five bioactive sponge-associated Pseudomonadaceae strains

**Table S6. Diversity of BGCs across the five bioactive sponge-associated Pseudomonadaceae strains**

| **Strain** | **Prediction tool** | **DeepBGC Score** | **Product class** | **Most similar known cluster (similarity %)** | **Cluster Blast similarity (%)** | **Predicted product activity** | **Gene cluster location** | | | **ARTS** | | |
| --- | --- | --- | --- | --- | --- | --- | --- | --- | --- | --- | --- | --- |
|  |  |  |  |  |  |  | **from** | **to** | **Size (nt)** | **Resistance markers** | | **Duplicated core gene** |
|  | DeepBGC | 0.78 | Other | - | 30 | Antibacterial | 79,661 | 92,78 | 13,12 | Resistance-nodulation-cell division (RND) antibiotic efflux pump [ARO:0010004] | | - |
|  | Antismash | - | NRPS-like | MA026 (7) | 77 |  | 55,565 | 91,413 | 35,849 | - |  | + |
|  | Antismash | - | RRE containing | - | 94 | - | 12,153 | 32,434 | 20,282 | - |  | + |
|  | DeepBGC | 0.72 | Other | - | 19 | - | 49,564 | 68,221 | 18,658 | - |  | - |
|  | DeepBGC | 0.95 | saccharide | Lipopolysaccharide (20) | 47 | - | 87 | 44,568 | 44,482 | - |  | + |
|  | DeepBGC | 0.58 | Polyketide | - | 10 | Antibacterial | 32,443 | 43,483 | 11,04 | - |  | + |
|  | DeepBGC | 0.55 | Terpene | 12-epi-hapalindole (6) | 15 | Antibacterial | 39,806 | 42,696 | 2,891 | - |  | - |
|  | DeepBGC and Antismash | 0.79 | NRP-metallophore, NRPS | Pyoverdine (41) | 44 | - | 1 | 36,084 | 36,084 | macB: subunit of efflux pump conferring antibiotic resistance [ARO:3000535] | | - |
| *Pseudomonas juntendi* 30M15 | DeepBGC | 0.70 | RiPP | - | 15 | Antibacterial | 232 | 27,215 | 26,984 | - |  | + |
|  | DeepBGC | 0.55 | Polyketide | - | 8 | Antibacterial, Antifung | 28,218 | 33,175 | 4,958 | - |  | - |
|  | DeepBGC | 0.77 | RiPP | Pyoverdine (3) | 38 | Antibacterial | 73 | 28,414 | 28,342 | - |  | + |
|  | DeepBGC | 0.58 | Polyketide | - | 22 | Antibacterial | 21,036 | 24,004 | 2,969 | - |  | - |
|  | DeepBGC | 0.77 | Polyketide | - | 39 | Antibacterial | 1 | 12,71 | 12,71 | Carboxyl transferase domain | | - |
|  | DeepBGC and Antismash | 0.72 | NRPS | Pyoverdine (5) | 37 | Antibacterial | 1 | 24,846 | 24,846 | - |  | + |
|  | BAGEL4 | - | RiPP | Zoocin A (42) | - | - | 15593 | 28346 |  | - |  | - |
|  | Antismash | - | NAGGN | - | 50 | - | 13,555 | 23,292 | 9,738 | - |  | - |
|  | Antismash | - | RiPP-like | - | 85 | - | 11,222 | 20,478 | 9,257 | - |  | + |
|  | DeepBGC | 0.98 | saccharide | Exopolysaccharide (16) | 24 | - | 60 | 20,1 | 19,941 | - |  | - |
|  | Antismash | - | NRPS, NRPS-like | Pyoverdine (5) | 14 | - | 1 | 17,539 | 17,539 | - |  | + |
|  | Antismash | - | Redox-cofactor | Lankacidin (13) | 62 | - | 1,972 | 16,034 | 14,063 | - |  | - |
|  | Antismash | - | NRPS | Pyoverdine (3) | 8 | - | 1 | 12,376 | 12,376 | - |  | + |
|  | DeepBGC and Antismash | 0.69 | NRPS-like | MA026 (7) | 96 | Antibacterial | 17,533 | 60,301 | 42,769 | - |  | - |
|  | DeepBGC | 0.75 | Polyketide | Lysocin (5) | 20 | - | 69,606 | 77,599 | 7,994 | - |  | - |
|  | DeepBGC | 0.97 | Saccharide | Lipopolysaccharide (14) | 39 | - | 16,401 | 59,7 | 43,3 | - |  | + |
|  | DeepBGC | 0.71 | Saccharide | - | 5 | Antibacterial | 40,444 | 54,476 | 14,033 | - |  | - |
|  | DeepBGC | 0.75 | Other | - | 19 | - | 147 | 19,887 | 19,741 | - |  | - |
|  | Antismash | - | RRE | - | 77 | - | 35,934 | 51,243 | 15,31 | - |  | - |
|  | DeepBGC | 0.56 | RiPP | - | 9 | Antibacterial | 36,552 | 40,515 | 3,964 | - |  | - |
|  | DeepBGC | 0.60 | Saccharide | Lipopolysaccharide (18) | - | - | 2,28 | 7,132 | 4,853 | - |  | - |
|  | DeepBGC | 0.55 | Polyketide | - | 47 | Antibacterial, Antifung | 28,227 | 33,184 | 4,958 | - |  | - |
|  | DeepBGC | 0.77 | RiPP | Pyoverdine (3) | 33 | Antibacterial | 133 | 28,474 | 28,342 | - |  | - |
| *Pseudomonas juntendi* 30B13 | Antismash | - | NAGGN | - | 45 | - | 13,502 | 23,239 | 9,738 | - |  | - |
|  | Antismash | - | NRPS | Pyoverdine (4) | 35 | Antibacterial | 1 | 23,096 | 23,096 | - |  | - |
|  | DeepBGC | 0.82 | Polyketide | - | 4 | Antibacterial | 237 | 2,893 | 2,657 | - |  | - |
|  | DeepBGC | 0.60 | Terpene | 12-epi-hapalindole (6) | 15 | Antibacterial | 15,914 | 20,817 | 4,904 | - |  | - |
|  | DeepBGC | 0.98 | Saccharide | Exopolysaccharide (16) | 21 | - | 160 | 20,1 | 19,941 | - |  | + |
|  | BAGEL4 | - | RiPP | Zoocin A (42) | - | - | 15,591 | 28,395 | 12,792 | - |  | - |
|  | Antismash | - | NRPS-like, NRPS | Pyoverdine (4) | 14 | Antibacterial | 1 | 18,832 | 18,832 | - |  | - |
|  | Antismash | - | NRPS, NRP-metallophore | Pyoverdine (29) | 25 | Antibacterial | 1 | 15,901 | 15,901 | - |  | - |
|  | Antismash | - | RiPP-like | - | 85 | - | 1 | 9,287 | 9,287 | - |  | - |
|  | Antismash | - | NRPS | Azotobactin (25) | 16 | - | 1 | 12,376 | 12,376 | - |  | - |
|  | Antismash | - | Redox-cofactor | Lankacidin c (13) | 25 | - | 1 | 4,214 | 4,214 | - |  | - |
| *Pseudomonas asiatica* 30BD33 | DeepBGC | 0.76 | Polyketide | Koreenceine (100) | 25 | Antibacterial | 46,665 | 64,583 | 17,919 | - |  | - |
|  | DeepBGC | 0.56 | saccharide | Lipopolysaccharide (18) | - | - | 2,279 | 4,94 | 2,662 | - |  | - |
|  | DeepBGC and Antismash | 0.83 | Redox-cofactor | lankacidin C (13) | 94 | - | 29,49 | 54,203 | 24,714 | - |  | - |
|  | DeepBGC | 0.71 | saccharide | - | 21 | - | 6,713 | 33,818 | 27,106 | msbA: ATP-binding cassette (ABC) antibiotic efflux pump [ARO:3000460] | | - |

Grey cells indicate BGCs with < 80% similarity with other known clusters of reference; - : Absence of information.

Continued…

**Table S6. Diversity of BGCs across the five bioactive sponge-associated Pseudomonadaceae strains**

| **Strain** | **Prediction tool** | **DeepBGC Score** | **Product class** | **Most similar known cluster (similarity %)** | **Cluster Blast similarity (%)** | **Predicted product activity** | **Gene cluster location** | | | **ARTS** | | |
| --- | --- | --- | --- | --- | --- | --- | --- | --- | --- | --- | --- | --- |
|  | DeepBGC | 0.58 | Polyketide | - | 5 | Antibacterial | 46,22 | 48,881 | 2,662 | - |  | - |
| *Pseudomonas asiatica* 30BD33 | DeepBGC | 0.67 | Polyketide | Alginate (41) | 21 | - | 3 | 10,178 | 10,176 | - | | - |
|  | DeepBGC | 0.83 | Saccharide | Viscosin (18) | 41 | - | 3 | 42,724 | 42,722 | - | | + |
|  | DeepBGC | 0.69 | Polyketide | - | 23 | - | 397 | 17,342 | 16,946 | - | | - |
|  | DeepBGC | 0.66 | Polyketide | - | - | Antibacterial, Antifungal | 4,716 | 12,822 | 8,107 | - | | - |
|  | DeepBGC | 0.59 | Other | - | 21 | Antibacterial | 34,196 | 38,231 | 4,036 | - | | + |
|  | Antismash | - | NRPS | Pyoverdine (4) | 47 | - | 1 | 36,727 | 36,727 | - | | - |
|  | DeepBGC | 0.91 | Saccharide | O-antigen (6) | 32 | - | 764 | 31,063 | 30,3 | - | | + |
|  | DeepBGC | 0.69 | RiPP | - | 14 | Antibacterial | 5,092 | 27,251 | 22,16 | - | | - |
|  | DeepBGC | 0.57 | Polyketide | - | 16 | Antibacterial | 13,855 | 22,001 | 8,147 | - | | - |
|  | DeepBGC | 0.58 | NRP+Polyketide | - | 10 | - | 17,579 | 19,638 | 2,06 | - | | - |
|  | Antismash and BAGEL4 | - | RiPP+Ranthipeptide | Pyoverdine (4) | 78 | - | 1,05 | 17,518 | 16,469 | Resistance-nodulation-cell division (RND) antibiotic efflux pump [ARO:0010004] | | - |
|  | Antismash | - | NRPS | Azotobactin (25) | 12 | - | 1 | 17,067 | 17,067 | - | | - |
|  | Antismash | - | RRE containing | - | 63 | - | 1 | 16,385 | 16,385 | Subclass B1 (metallo-) beta-lactamase hydrolize penicillins, cephalosporins and carbapenems [ARO:3000568] | | - |
|  | Antismash | - | RiPP-like | - | 50 | - | 6,301 | 13,623 | 7,323 | - | | - |
|  | Antismash | - | NRPS, NRP-metallophore | Pyoverdine (12) | 16 | - | 1 | 13,16 | 13,16 | - | | - |
|  | Antismash | - | RiPP-like | - | 44 | - | 1 | 6,664 | 6,664 | - | | - |
|  | Antismash | - | NRPS | Taiwachelin (11) | 8 | - | 1 | 6,32 | 6,32 | - | | - |
|  | Antismash | - | NRPS-like | Icosalide (100) | - | - | 1 | 1,156 | 1,156 | - | | - |
| *Ectopseudomonas khazarica*  30M25 | DeepBGC | 0.83 | Saccharide | Lipopolysaccharide (27) | 12 | - | 261,62 | 280,472 | 18,853 | - | | - |
|  | DeepBGC | 0.83 | Saccharide | Lipopolysaccharide (27) | 12 | - | 261,62 | 280,472 | 18,853 | - | | - |
|  | DeepBGC and Antismash | 0.87 | NRP-metallophore, NRPS, NI-siderophore | Azotobactin (66) | 100 | - | 162,299 | 270,379 | 108,081 | - | | - |
|  | Antismash | - | RiPP-like | - | 100 | - | 265,671 | 277,869 | 12,199 | - | | - |
|  | DeepBGC and Antismash | 0.56 | RiPP-like | - | 100 | - | 239,05 | 249,892 | 10,843 | - | | - |
|  | Antismash | - | RiPP-like | - | 90 | - | 167,673 | 178,509 | 10,837 | - | | - |
|  | DeepBGC | 0.63 | Saccharide | Alginate (50) | 20 | - | 123,173 | 131,432 | 8,26 | - | | - |
|  | DeepBGC | 0.70 | Polyketide | Lysocin (5) | 22 | - | 170,815 | 177,397 | 6,583 | - | | - |
|  | Antismash | - | NAGGN | - | 77 | - | 178,753 | 189,582 | 10,83 | - | | - |
|  | DeepBGC and Antismash | 0.79 | Betalactone, Ranthipeptide | Pyoverdine (8) | 100 | Antibacterial | 120,804 | 160,349 | 39,546 | Biotin-requiring enzyme | | - |
|  | DeepBGC and Antismash | 0.56 | RiPP-like | - | 100 | - | 61,271 | 72,113 | 10,843 | - | | - |
|  | DeepBGC | 0.94 | Saccharide | O-antigen (14) | 25 | - | 372 | 19,81 | 19,439 | - | | - |
|  | DeepBGC | 0.60 | Saccharide | - | 6 | - | 360 | 14,011 | 13,652 | - | | - |
|  | Antismash | - | Redox-cofactor | Lankacidin c (13) | 63 | - | 5,608 | 19,285 | 13,678 | - | | - |

Grey cells indicate BGCs with < 80% similarity with other known clusters of reference; - : Absence of information.

Continued…

**Table S6. Diversity of BGCs across the five bioactive sponge-associated Pseudomonadaceae strains**

| **Strain** | **Prediction tool** | **DeepBGC Score** | **Product class** | **Most similar known cluster (similarity %)** | **Cluster Blast similarity (%)** | **Predicted product activity** | **Gene cluster location** | | | **ARTS** | |
| --- | --- | --- | --- | --- | --- | --- | --- | --- | --- | --- | --- |
|  | DeepBGC | 0.84 | Saccharide | Lipopolysaccharide (27) | 12 | - | 207,032 | 225,893 | 18,862 | msbA: ATP-binding cassette (ABC) antibiotic efflux pump [ARO:3000460] | - |
| *Pseudomonas asiatica* 30BD33 | DeepBGC | 0.62 | Other | Pederin/pseudopederin/pederone (12) | 44 | Antibacterial | 123,906 | 132,378 | 8,473 | - | + |
|  | DeepBGC AND Antismash | 0.52 | Betalactone | Fengycin (26) | 92 | Antibacterial | 138,622 | 173,936 | 35,315 | Carboxyl transferase domain | - |
|  | DeepBGC | 0.57 | NRP+Polyketide | - | 14 | Antibacterial | 215,88 | 218,998 | 3,119 | - | + |
|  | DeepBGC AND Antismash | 0.81 | NRP-metallophore, NRPS | Pyoverdine (22) | 65 | - | 1 | 38,231 | 38,231 | - | - |
|  | Antismash | - | RiPP-Like | - | 81 | - | 42,212 | 53,048 | 10,837 | - | - |
|  | Antismash | - | RiPP-Like | - | 100 | - | 86,308 | 97,15 | 10,843 | - | - |
|  | DeepBGC | 0.92 | Saccharide | O-antigen (38) | 31 | Cytotoxic | 10,187 | 48,632 | 38,446 | - | + |
|  | DeepBGC and Antismash | 0.57 | RiPP-Like | - | 100 | Antibacterial | 48,438 | 59,28 | 10,843 | - | - |
|  | Antismash | - | RiPP-Like | Iturin (22) | 88 | - | 1 | 9,778 | 9,778 | - | - |
|  | DeepBGC AND Antismash | 0.90 | NRP-metallophore, NRPS | Azotobactin (41) | 55 | - | 1 | 57,003 | 57,003 | - | - |
|  | DeepBGC | 0.60 | Saccharide | Alginate (50) | 20 | Antibacterial | 15,203 | 23,612 | 8,41 | - | - |
|  | Antismash | - | NAGGN | - | 77 | - | 1 | 10,774 | 10,774 | - | + |
|  | DeepBGC AND Antismash | 0.54 | Redox-cofactor | Lankacidin C (13) | 57 | Antibacterial | 23,095 | 36,763 | 13,669 | - | + |
|  | Antismash | - | NRPS-Like | Cyanopeptolin (75) | 15 | - | 1 | 2,418 | 2,418 | - | - |

Grey cells indicate BGCs with < 80% similarity with other known clusters of reference; - : Absence of information.
